# Milton assembles large mitochondrial clusters, mitoballs, to sustain spermatogenesis

**DOI:** 10.1073/pnas.2306073120

**Published:** 2023-08-14

**Authors:** Andy Y. Z. Li, Ying Di, Sumaera Rathore, Ason C.-Y. Chiang, Jan Jezek, Hansong Ma

**Affiliations:** ^a^Wellcome/Cancer Research UK Gurdon Institute, Cambridge CB2 1QN, United Kingdom; ^b^Department of Genetics, University of Cambridge, Cambridge CB2 3EH, United Kingdom; ^c^School of Biosciences, University of Birmingham, Birmingham B15 2TT, United Kingdom

**Keywords:** mitochondrial clustering, insect spermatogenesis, Milton-mediated mitochondrial trafficking, mitoballs, male fertility

## Abstract

Mitochondria are dynamic organelles that undergo frequent remodeling to accommodate developmental needs. Here, we describe a striking organization of mitochondria into a large ball-like structure adjacent to the nucleus in premeiotic *Drosophila melanogaster* spermatocytes, which we term “mitoball”. Mitoballs are transient structures that colocalize with the endoplasmic reticulum, Golgi bodies, and the fusome. We observed similar premeiotic mitochondrial clusters in a wide range of insect species, including mosquitos and cockroaches. Through a genetic screen, we identified that Milton, an adaptor protein that links mitochondria to microtubule-based motors, mediates mitoball formation. Flies lacking a 54 amino acid region in the C terminus of Milton completely lacked mitoballs, had swollen mitochondria in their spermatocytes, and showed reduced male fertility. We suggest that the premeiotic mitochondrial clustering is a conserved feature of insect spermatogenesis that supports sperm development.

## Significance

This work unveils the intriguing phenomenon of large mitochondrial clusters (mitoballs) formed during insect spermatogenesis. It offers unparalleled insights into the dynamic nature of mitochondria and their remarkable ability to adapt to developmental needs. The identification of Milton’s role in mitoball formation and mitochondrial function prompts further exploration of its involvement in human male fertility, given the conserved function of insect and human Milton in mitochondrial trafficking. Moreover, our findings lay the foundation for future investigations connecting subcellular mitochondrial distribution to mitochondrial function and male fertility.

**Fig. 1.**
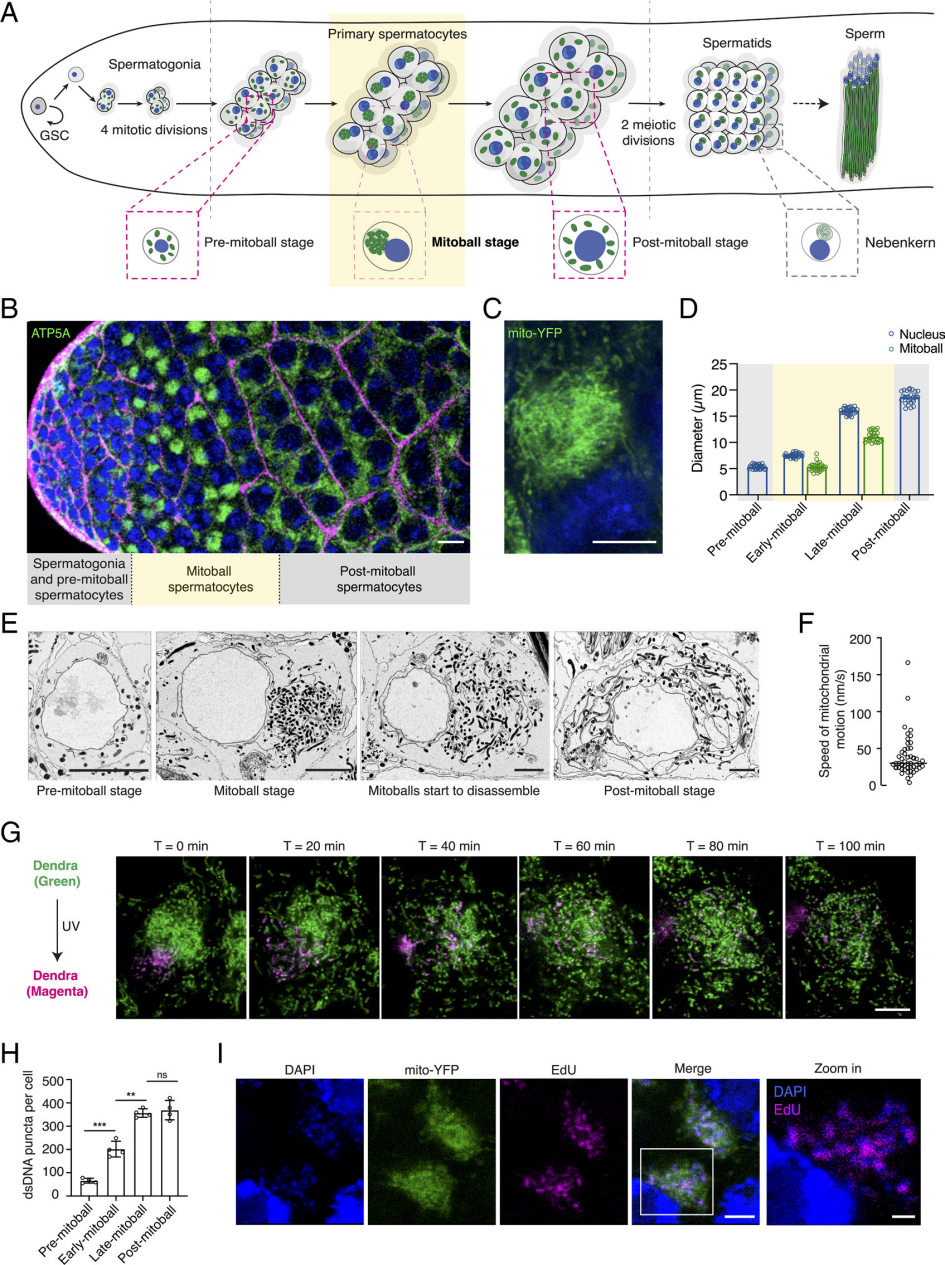
Mitoballs are transient structures that assemble and disassemble during prophase I of *Drosophila* spermatogenesis. (*A*) Illustration of *D. melanogaster* spermatogenesis. The germline stem cells (GSCs) are contained at the apical tip of the testis. Each GSC undergoes numerous asymmetric cell divisions and each time, it self-renews and produces a gonialblast. The gonialblast then goes through four mitotic divisions to form 16 spermatogonia cells interconnected by the fusome (known as a cyst) due to incomplete cytokinesis. The premeiotic S phase occurs quickly after the last mitotic division, and the bulk of primary spermatocyte development occurs in a long premeiotic G2 phase of the cell cycle, which increases the cell size by ~25 times (9). This is followed by two meiotic divisions to generate 64 spermatids, sperm elongation, and maturation (10). Mitoballs form in the primary spermatocytes and nebenkerns form in early spermatids, prior to sperm elongation (*SI Appendix*, Fig. S1 *A* and *B*). Mitochondria and nuclei are labeled in green and blue, respectively. Drawings are not to scale. (*B*) The apical tip of a *D. melanogaster* testis showing germ cells in mitotic and prophase I stages. The testis was stained with DAPI (blue), anti-ATP5A antibodies (green) and phalloidin (magenta) to visualize DNA, mitochondria, and cell/cyst boundaries, respectively. (Scale bar: 20 μm.) (*C*) A zoomed-in view of a mitoball in sqh-mito-YFP flies. (Scale bar: 5 μm.) (*D*) The diameters of nuclei and mitoballs of spermatocytes at different stages (n = 28). Error bars: SDs. (*E*) Electron microscopic images of spermatocytes at premitoball, mitoball and postmitoball stages. (Scale bars: 5 μm.) (*F*) The speed of mitochondrial motion in mitoballs (n = 48) (Movie S1). (*G*) Time-lapse images illustrating the dynamics of mitochondria within mitoballs. The expression of mito-Dendra2 in early germ cells was driven by bamGAL4. A subpopulation of mito-Dendra2 was converted from the green to red fluorescent form and followed for 100 min. (Scale bar: 5 μm.) (*H*) The total mtDNA copy number in spermatocytes of different stages (n = 4). Antibodies against dsDNA were used to stain mtDNA nucleoids and count the total mtDNA copy number per cell (*SI Appendix*, Fig. S1*E*). Error bars: SDs, one-way ANOVA and Tukey’s post hoc, ns *P* > 0.05, **P < 0.01, ***P < 0.005. (*I*) EdU staining reveals active mtDNA replication during the mitoball stage (*SI Appendix*, Fig. S1*F*). [Scale bars: 5 μm (1 μm for the zoomed-in view).] Testes were dissected from sqh-mito-YFP (green), incubated with EdU (magenta) for 4 h, fixed, and stained with DAPI (blue).

The mitochondrial network changes its organization in response to developmental and environmental cues (1, 2). Spermatogenesis is a complex differentiation process with cascades of tightly regulated transitions in cell states. These transitions are often coupled with the dynamic rearrangement of the mitochondrial network. In *Drosophila*, one of the most striking mitochondrial transformations occurs after the completion of meiosis in the primary spermatids, where hundreds of mitochondria fuse into two long mitochondrial derivatives that wrap around each other to form a spherical aggregate adjacent to the nucleus. This is called the nebenkern, which has been known since the 19th century and is widely conserved in insects (3) (Fig. 1*A* and *SI Appendix*, Fig. S1 *A* and *B*). The nebenkern disassembles as the two mitochondrial derivatives unfold and extend beside the growing axoneme during flagellar elongation (4–6). Since the discovery of the nebenkern, there is increasing evidence that mitochondrial distribution and organization are crucial for germ cell differentiation and development (1, 7, 8). However, little is known about the molecular mechanisms regulating mitochondrial network organization and how they impact sperm function and male fertility.

Here, we described another dramatic reorganization of the mitochondrial network that occurs during the mitosis-to-meiosis transition in *Drosophila melanogaster* spermatogenesis. We named this organization “mitoball” because mitochondria in each spermatocyte cluster to form a large ball-like structure next to the nucleus. These structures were first observed in 1971 through a comprehensive electron microscopy study of the *D. melanogaster* testis (4), but their properties and dynamics remained uncharacterized. In this study, we describe the formation and disassembly of mitoballs and show that Milton-dependent mitochondrial trafficking is required for their formation. The loss of mitoballs in hypomorphic *milton* mutants is associated with abnormal mitochondrial morphology and reduced male fertility. Importantly, we show that premeiotic mitochondrial clusters are found in a range of insect species, indicating that they are a general hallmark of insect spermatogenesis.

## Results

### Mitoballs Are Transient Structures That Assemble and Disassemble during the Prophase of Meiosis I.

We constantly observed mitoballs in larval and adult primary spermatocytes (Fig. 1 *B* and *C* and *SI Appendix*, Fig. S1 *A* and *C*). Like in humans and mice, *Drosophila* spermatogenesis consists of four key steps: 1) differentiation of germline stem cells into spermatogonia cells, 2) mitotic divisions of spermatogonia cells to give rise to primary spermatocytes, 3) meiotic divisions to generate haploid spermatids, and 4) spermatid elongation and maturation (Fig. 1*A*) (10). In *D. melanogaster*, it is known that after the last mitotic division, the premeiotic S phase initiates and completes very quickly, and early primary spermatocytes are similar in size to postmitotic spermatogonial cells (10, 11) (with a nuclear diameter of ~5 μm, Fig. 1*D*). We found that as primary spermatocytes entered the premeiotic G2 phase (corresponds to prophase I), which is a growth phase that lasts for ~80 h (9), mitoballs began to form (Fig. 1*B*). The nucleus progressively increased in size, and the three chromatin clumps (*SI Appendix*, Fig. S1*D*), due to the separation of paired sister chromatids, became apparent (9, 11). When the nucleus reached ~7.5 μm, round-shaped mitoballs of ~5 μm were clearly visible (Fig. 1*D*). Mitoballs continued to grow and eventually reached ~11 μm in diameter before mitochondria started to disperse uniformly into the cytoplasm, and the nucleus progressively resumed a central position within the cell (Fig. 1 *B* and *D*). The entire mitoball stage lasted at least 8 h based on our time-lapse live imaging.

We confirmed the mitochondrial dynamics from the premitoball to postmitoball stages by electron microscopy imaging (Fig. 1*E*). Unlike the nebenkern, mitochondria within the mitoball were not fused. They were highly dynamic rod-shaped individuals, which constantly moved in all directions at an average speed of ~30 nm/s (Fig. 1*F* and Movie S1). By expressing mitochondrially targeted Dendra2 (mito-Dendra2), a monomeric fluorescent protein that can be irreversibly photoconverted from an initial green fluorescent form to a red fluorescent form by ultraviolet (UV) light, we converted and tracked a subpopulation of mitochondria within a mitoball. We found that most converted mitochondria (labelled as magenta in Fig. 1*G*) stayed as discrete populations after 100 min, despite continuously relocating within the mitoball. This result indicates a low level of exchange among the mitochondrial population within the same mitoball.

The gradual increase in mitoball size suggests that mitochondrial biogenesis occurs during this period. To examine whether mitochondrial DNA (mtDNA) is also being replicated during this stage, we quantified the number of mtDNA foci in primary spermatocytes before, during, and after the mitoball stage by staining testes with anti-dsDNA antibodies (*SI Appendix*, Fig. S1*E*). Prior to mitoball formation, the average mtDNA nucleoid copy number was ~65 in each spermatocyte. The number increased to ~200 when the mitoballs were ~5 μm in diameter. It eventually reached ~360 in the late mitoball stage and remained approximately the same in postmitoball spermatocytes (Fig. 1*H*).

Our quantification using dsDNA antibody staining might not reflect the actual increase in mtDNA copy number as individual dots could represent a single mtDNA nucleoid or multiple nucleoids in close proximity. Hence, we further examined mtDNA replication by EdU staining. We observed clear EdU puncta in mitochondria at the mitotic and mitoball stages, but no signal was detected after the mitoball stage even with extended incubation (16 h) with EdU (Fig. 1*I* and *SI Appendix*, Fig. S1*F*). Strong mtDNA EdU staining was only observed again at the nebenkern stage (*SI Appendix*, Fig. S1*F*). In line with this observation, the expression level of the endogenous mtDNA polymerase *PolG1* was high in mitotic cells and spermatocytes at the mitoball stage, and much lower in postmitoball spermatocytes (*SI Appendix*, Fig. S1*G*). This suggests that mtDNA replication in early spermatogenesis predominantly occurs before mitoball disassembly. Taken together, we conclude that mitoballs are a collection of dynamic mitochondria that form a spherical cluster on one side of primary spermatocytes in early prophase I and disperse as the cells progress into the later period of this phase. The mitoball stage is also coupled with mitochondrial biogenesis and mtDNA replication.

### Premeiotic Mitoballs and Similar Mitochondrial Clusters Were Found in Many Other Insect Species.

**Fig. 2.**
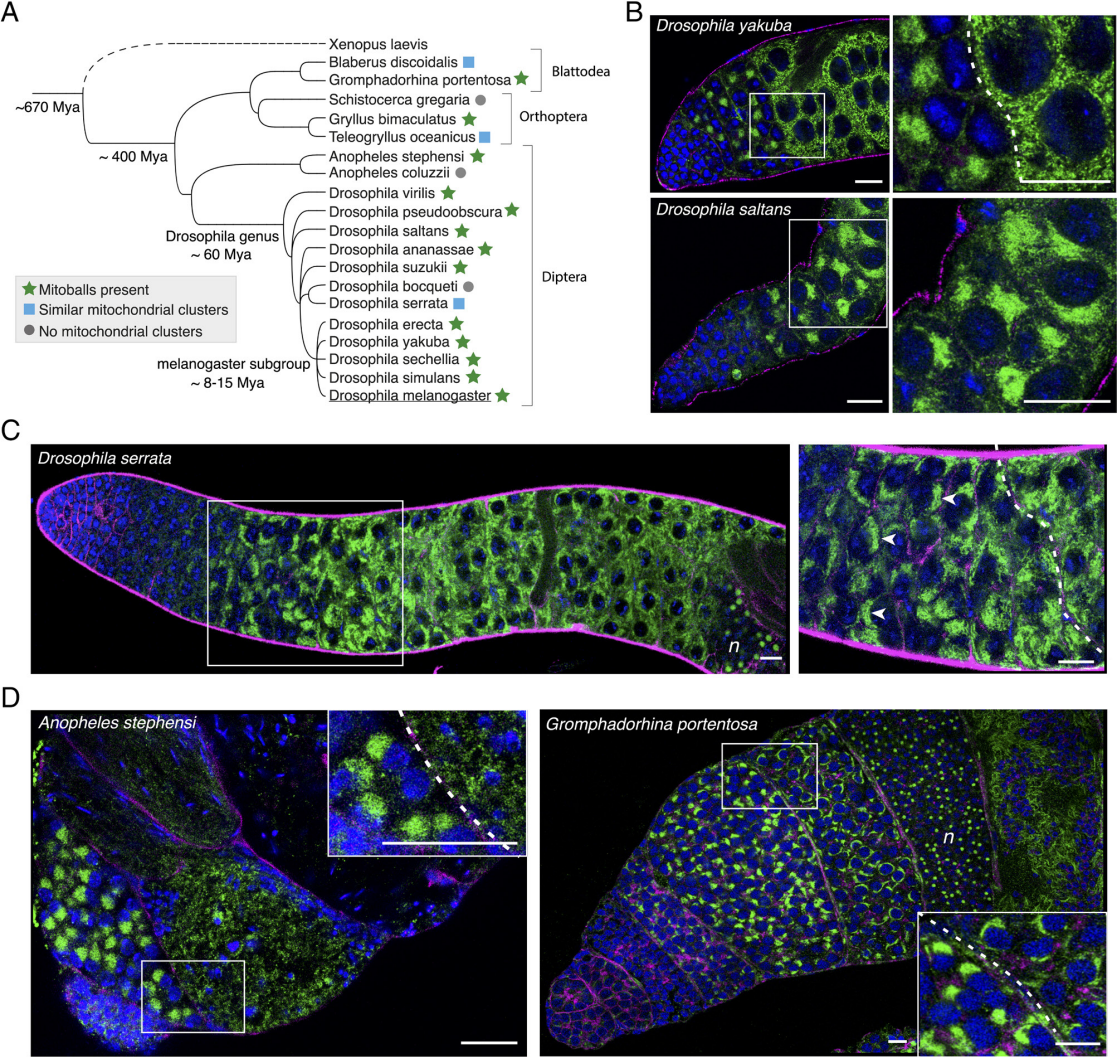
Mitoballs and similar mitochondrial clusters also form in other insect species. (*A*) A phylogenetic tree listing insect species examined in this study for the presence of mitoballs or similar mitochondrial clusters in spermatocytes. The phylogenetic relationships were based on NCBI datasets and created using PhyIoT. The divergence times were based on previous reports (12–14). (*B*) Representative images of mitoballs in two *Drosophila* species. (*C*) The premeiotic mitochondrial clusters observed in *D. serrata* spermatocytes were more crescent-shaped (examples are indicated by white arrowheads in the zoomed-in view). The letter ‘*n*’ indicates the nebenkern region. (*D*) Representative images of the apical tips of *Anopheles stephensi* and *Gromphadorhina portentosa* testes. The letter ‘*n*’ indicates the nebenkern region. For all samples, testes were stained with DAPI (blue), anti-ATP5A antibodies (green) and phalloidin (magenta) to visualize DNA, mitochondria, and cell/cyst boundaries, respectively. Cysts at the mitoball and postmitoball stages are separated by a dotted line in the zoomed-in views. (All the scale bars: 20 μm.)

The nebenkern structure is found in many insect species (3). We wondered whether the same applied to mitoballs. By immunostaining, we found mitoball-like structures in all *Drosophila* species of the *melanogaster* subgroup, and many distantly related *Drosophila* species such as *Drosophila saltans* and *Drosophila pseudoobscura* (Fig. 2 *A* and *B* and *SI Appendix*, Fig. S2*A*). Mitochondrial clusters were also observed in spermatocytes of *Drosophila serrata*, but these clusters were more crescent-shaped rather than spherical (Fig. 2*C*). Nevertheless, they followed a similar dynamic to mitoballs: they formed during the early premeiotic G2 phase, and the mitochondria dispersed in the cytoplasm as cells progress into a later period of this phase (Fig. 2*C*). *Drosophila bocqueti*, on the other hand, did not seem to have mitoballs, but nebenkerns were observed (*SI Appendix*, Fig. S2*B*).

We then examined two mosquito species that are malaria vectors. Like *Drosophila*, they belong to the order Diptera. Clear mitoballs were observed in *Anopheles stephensi* (Fig. 2*D*), but not in the closely related *Anopheles coluzzii* (*SI Appendix*, Fig. S2*B*). Interestingly, neither species seemed to have nebenkerns. We also examined five species in the orders of Orthoptera (locusts and crickets) and Blattodea (cockroaches), which diverged from Diptera about 400 Mya. Mitoball structures were visible in one cockroach (*Gromphadorhina portentosa*) and one cricket species (*Gryllus bimaculatus*) (Fig. 2*D* and *SI Appendix*, Fig. S2*A*). In the other cockroach (*Blaberus discoidalis*) and cricket (*Teleogryllus oceanicus*) species, mitochondria also formed clusters in the spermatocytes that followed a similar dynamic to mitoballs but, as in *Drosophila serrata* (Fig. 2*C*), these clusters were more crescent-shaped (*SI Appendix*, Fig. S2*C*). For the locust species, *Schistocerca gregaria*, no mitoballs were observed while nebenkerns were apparent (*SI Appendix*, Fig. S2*B*). These findings show that mitochondrial clustering in spermatocytes is conserved across a wide range of insect species, but the structure and organization can vary even between closely related species.

### Mitoballs Are Packed with Endoplasmic Reticulum (ER), Surrounded by Golgi Bodies and in Direct Contact with the Fusome.

**Fig. 3.**
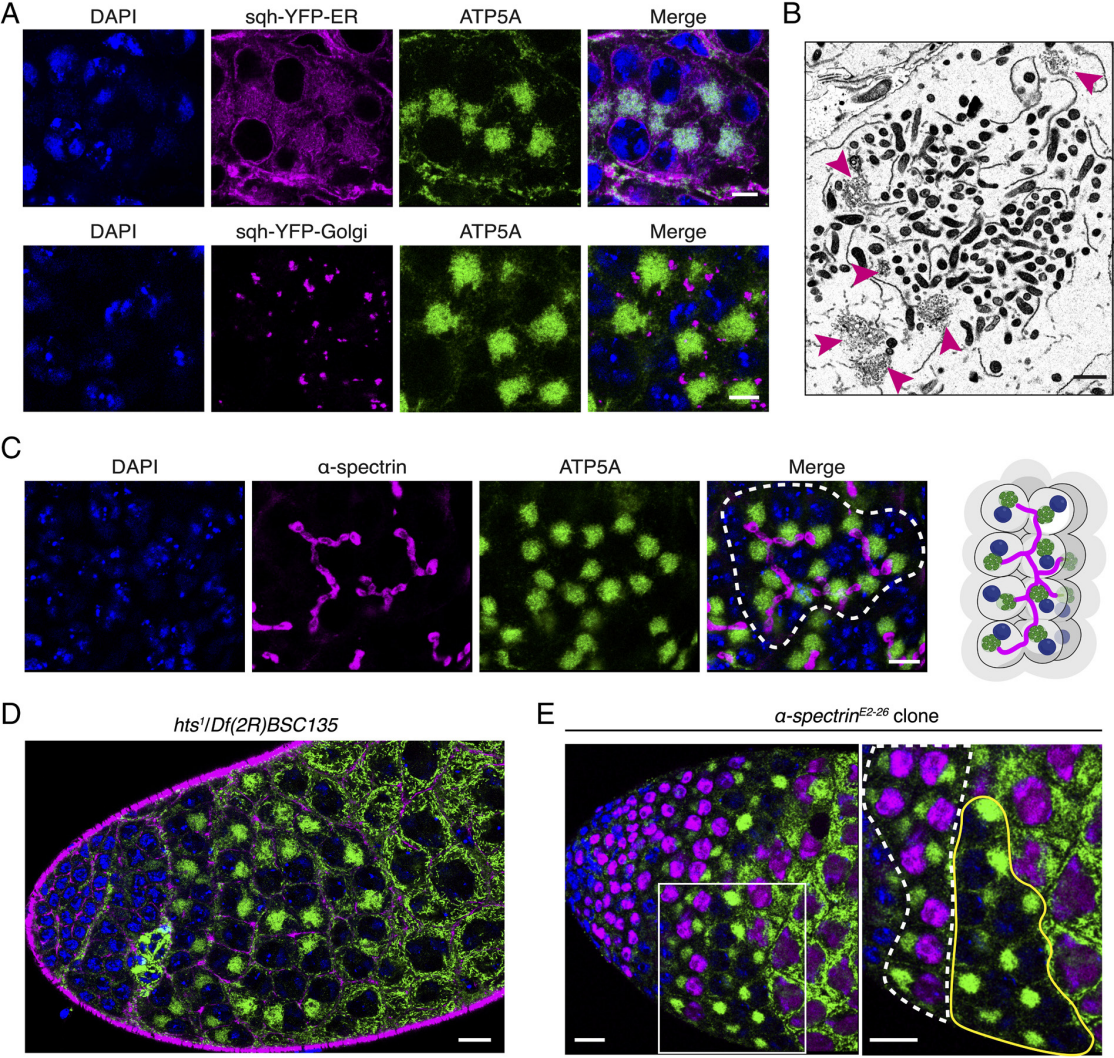
*D. melanogaster* mitoballs are packed with ER, surrounded by Golgi bodies, and connected by the fusome. (*A*) The distribution of the ER network and Golgi bodies in mitoball-stage spermatocytes. The ER and Golgi bodies (magenta) were visualized in sqh-YFP-ER (KDEL) and sqh-YFP-Golgi flies, respectively. Testes were stained with DAPI (blue) and anti-ATP5A antibodies (green). (Scale bars: 10 μm.) (*B*) An electron microscopic image showing that ER intertwines with mitochondria within the mitoball region, while Golgi bodies (pink arrowheads) are mostly located on the periphery. (Scale bar: 1 μm.) (*C*) The mitoball is adjacent to the fusome in each spermatocyte. (*Left*) A single cyst is outlined by dotted white lines in the merged image. The testis was stained with DAPI (blue), anti-ATP5A antibodies (green), and anti-α-spectrin antibodies (magenta). (Scale bar: 10 μm.) (*Right*) An illustration showing the relative position of nuclei, the fusome, and mitoballs in a cyst (see Movie S2 for a three-dimensional projection of the fusome and mitoballs in a cyst). (*D*) Mitoballs were observed in the *hts* mutant, *hts^1^*/*Df(2R)BSC135,* that lacks the fusome (*SI Appendix*, Fig. S3*E*). In line with previous studies (e.g., ref. 17), compromising the fusome did not cause abnormalities in testis morphology. The testis was stained with DAPI (blue), anti-ATP5A antibodies (green) and phalloidin (magenta). (Scale bar: 20 μm.) (*E*) Cysts that lack α-spectrin carried mitoballs. The *α-spectrin* knockout (KO) clone and a wild-type cyst are outlined by solid yellow and dotted white lines in the zoomed-in view, respectively. The testis was stained with DAPI (blue) and anti-ATP5A antibodies (green). KO cells were marked by the absence of RFP (magenta) in their nuclei. (Scale bars: 20 μm.)

Mitochondrial function and dynamics are modulated through close interactions with other organelles in the cell (15). To visualize whether mitoballs contain other organelles, we imaged *D. melanogaster* lines expressing fluorescent proteins targeted to either the ER or Golgi. Ubiquitous expression of YFP-KDEL showed that the ER network occupied a larger cytoplasmic region than the mitoballs, but they were heavily condensed in the mitoball area (Fig. 3*A*). This observation was validated using flies expressing GFP-HDEL in spermatocytes under bamGAL4 (*SI Appendix*, Fig. S3*A*). To visualize Golgi bodies, we used an sqh-YFP-Golgi line, which expresses YFP fused with the Golgi targeting sequence of the B4GALT1 gene under a ubiquitous promoter (16), and found that Golgi bodies mainly decorated the periphery of mitoballs (Fig. 3*A*). Electron microscopic images confirmed that the ER intertwined with mitochondria within mitoballs, while Golgi bodies were mainly located in the peripheral regions (Fig. 3*B*).

Another important organelle for germ cells is the fusome, which branches through the ring canals and connects the germ cells in each cyst (Fig. 3*C*). The fusome contains several cytoskeletal proteins including Adducin (coded by *hts* in *D. melanogaster*) and α-spectrin (17–20). During early oogenesis, mitochondria in cyst cells migrate along the fusome, and that facilitates the delivery of mitochondria from the nurse cells to the future oocyte (21). Our live imaging did not detect mitochondrial movement between spermatocytes via the fusome. However, the fusome was in direct contact with the mitoball in each spermatocyte, with the nucleus localizing to the other side (see Movie S2 and *SI Appendix*, Fig. S3*B* for the relative positions of the fusome, mitoballs, nuclei and ring canals in a cyst). In fact, when only mitochondria were visualized, the fusome could be identified within the mitoball by what resembles a cavity, devoid of any mitochondria, under certain focal planes (*SI Appendix*, Fig. S3*C*). Close contact with the fusome was observed for mitoball-like structures in many other species we examined, such as *A. stephensi* (*SI Appendix*, Fig. S3*D*). In summary, mitoballs are densely packed with ER, surrounded by Golgi bodies, and connected by the fusome within a cyst.

### Milton Is Required for Mitoball Formation.

Given the close contact of the fusome and mitoballs, we wondered whether the fusome was required for mitoball formation. To test this, we examined mitoballs in mutants of two genes required for fusome formation: *hts* and *α-spectrin*. Despite the absence of the fusome (*SI Appendix*, Fig. S3*E*), the mitoball appeared normal in the *hts^1^*/*Df(2R)BSC135* mutant (Fig. 3*D*) and *α-spectrin* knockout (KO) cysts (Fig. 3*E*). Hence, the fusome is dispensable for mitoball formation.

**Fig. 4.**
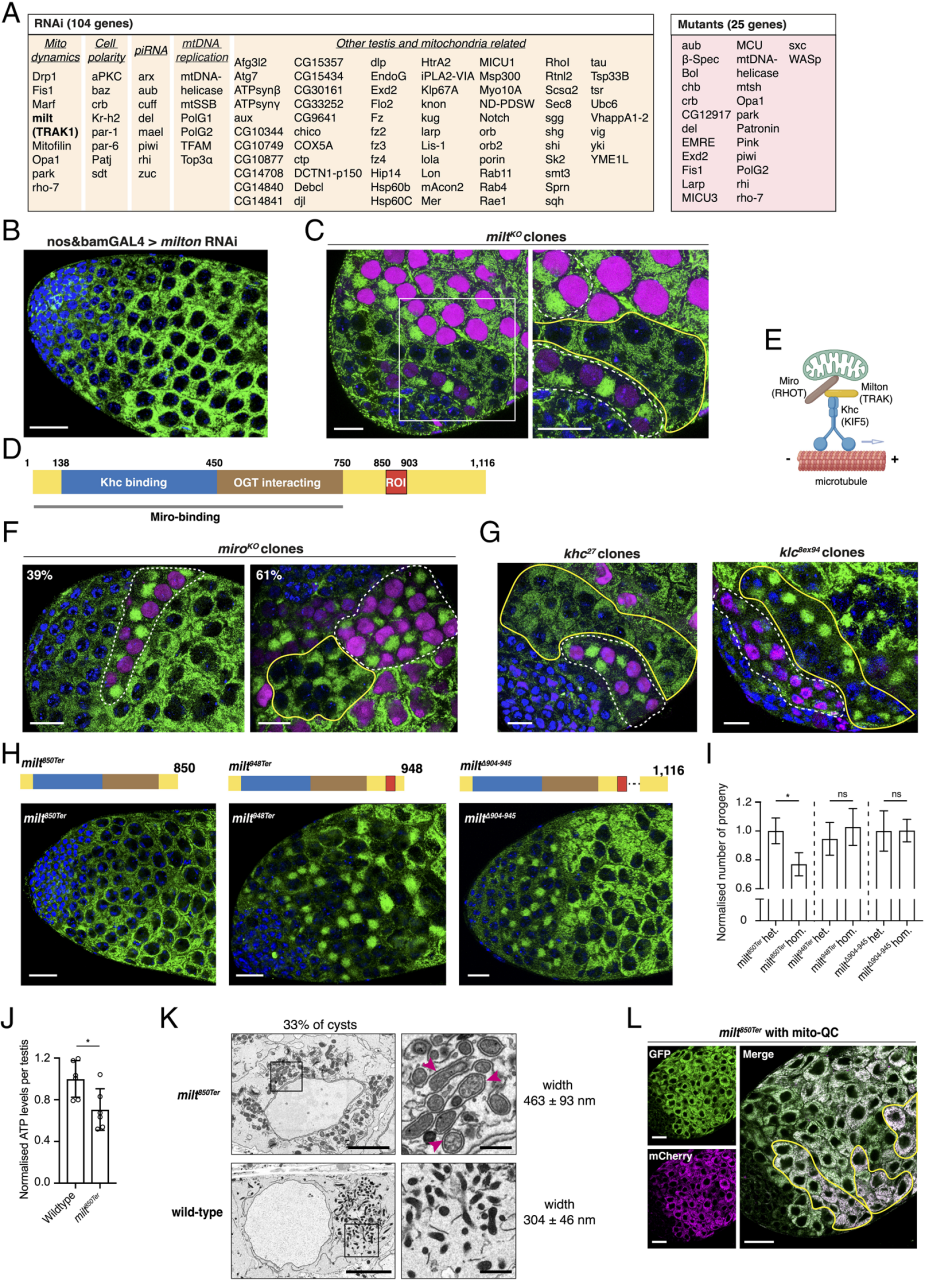
A candidate screen revealed that Milton is required for mitoball formation to sustain mitochondrial function and male fertility. (*A*) A list of 115 genes knocked down (RNAi) or knocked out (mutants) to examine their effect on mitoball formation. (*B*) No mitoballs were found in testes expressing *milton*-RNAi driven by nosGAL4 and bamGAL4. Mitochondria (green) were visualized by anti-ATP5A antibody staining. (Scale bar: 50 μm.) (*C*) Germline cysts that lack *milton* had no mitoballs. Control cysts (outlined by dotted white lines in the zoomed-in view) at a similar stage carry mitoballs. *milton* KO cells were marked by the absence of RFP (magenta) in their nuclei. (Scale bars: 20 μm.) (*D*) The function of Milton protein regions based on previous studies: the AA138-450 region is highly conserved and binds to Khc (23); the AA450–750 region binds to, and is a substrate for the cytosolic glycosylating enzyme *O*-GlcNAc transferase (OGT) (24); ROI: region of interest identified in this study to be essential for mitoball formation. (*E*) A schematic illustration of the Milton–Miro–Khc complex in transporting mitochondria along microtubules. The protein names for the human orthologs are in brackets. (*F*) Knocking out *miro* compromised mitoball formation. *miro* KO cysts had either no mitoballs (39%) or partial mitochondrial clustering (61%). (*Left*) A single wild-type cyst (outlined by dotted white lines) is surrounded by multiple KO cysts with no mitoballs. (*Right*) A KO cyst (outlined by solid yellow lines), next to a control cyst (outlined by dotted white lines), shows partial mitochondrial aggregation. (Scale bars: 20 μm.) (*G*) Mitoballs were found in *klc* KO cysts, but not in *khc* KO cysts. Mutant and control cyst clones are outlined by solid yellow and dotted white lines, respectively. (Scale bars: 20 μm.) (*H*) Mitoballs were only observed in flies containing the AA850–903 region of Milton. (Scale bars: 20 μm.) (*I*) Homozygous *milt^850Ter^* mutants with no mitoball showed reduced male fertility (n = 20). The numbers of progeny produced at 25 °C by homozygous mutants were normalized to the respective heterozygotes. Error bars: SEM, Mann–Whitney *U* test, ns *P* > 0.05, *P < 0.05. (*J*) The relative ATP level in testes of *milt^850Ter^* and wild-type controls (n = 6). Error bars: SDs, Student’s *t* test, *P < 0.05. (*K*) Electron micrographs illustrating a *milt^850Ter^* spermatocyte at the mitoball stage with no mitoballs, but abnormally swollen mitochondria surrounded by a layer of membrane reminiscent of ER. The ER-like membrane is indicated by pink arrowheads for some mitochondria in the zoomed-in view. [Scale bar: 5 μm (1 μm for the zoomed-in views).] (*L*) Representative images of mito-QC expression in a *milt^850ter^* testis. Some cysts with more mCherry-only foci are outlined. (Scale bars: 20 μm.)

To identify factors required for mitoball formation, we screened mutants and/or RNAi knockdowns (KD) of 115 candidate genes in early germline cells using nosGAL4 and BamGAL4 (Fig. 4*A* and *SI Appendix*, Tables S1 and S2). These genes encode proteins highly expressed in primary spermatocytes (22) or involved in relevant processes, such as mitochondrial dynamics, mtDNA replication, or cell polarity. Through our screen, we found that knocking down/out genes known to regulate mitochondrial morphology including *marf*, *opa1*, *MICU3* and *rho-7* caused defects in mitochondrial morphologies and/or testis development; however, mitoballs were still formed in many of these KD/KO lines (*SI Appendix*, Tables S1 and S2 and Fig. S4*A*). Knocking down *milton*, on the other hand, completely abolished mitoballs without causing any other obvious abnormalities in mitochondrial or testis morphology. The *milton* RNAi flies had mitochondria distributed evenly in the cytoplasm of the primary spermatocytes where the mitoball normally presents (Fig. 4*B* and Movie S3). To confirm the role of Milton in mitoball formation, we isolated two null mutants that carry premature stop codons near the N terminus by CRISPR/Cas9-based editing (*milt^KO1^* and *milt^KO2^*, *SI Appendix*, Fig. S4*B*). *milt^KO^* mutants were homozygous lethal, so we generated KO cyst clones in heterozygous flies. We found that *milt* KO cysts completely lacked mitoballs, whereas neighboring wild-type cysts at a similar developmental stage had clear mitoballs (Fig. 4*C* and *SI Appendix*, Fig. S4*C*). Hence, Milton is required for mitoball formation.

Milton is an adaptor protein that links mitochondria to microtubules, which exhibit a dense network in spermatocytes at the mitoball stage (*SI Appendix*, Fig. S4*D*). It was first identified in *D. melanogaster* for its role in recruiting kinesin heavy chain (Khc) to mitochondria and mediating their anterograde movement in axons (23, 25). Milton contains a Khc-binding domain near its N terminus (136 to 450) (Fig. 4*D*) (23). Its association with mitochondria is mediated by Miro, which is a Rho-like GTPase that locates in the outer membrane of mitochondria (Fig. 4*E*) (23, 26). Miro binds to the N terminus of Milton (1 to 750), and a dominant negative Miro that lacks its mitochondrial transmembrane domain can displace Milton from mitochondria (23). In humans, the Milton ortholog proteins TRAK1 and TRAK2 also form a complex with Miro (RHOT1/2) to mediate microtubule-based mitochondrial trafficking in neurons and other cell types (24, 27). To test whether Miro is also required for mitoball formation, we isolated two CRISPR mutants with premature stop codons near the N terminus (*SI Appendix*, Fig. S4*B*) and generated KO cyst clones in heterozygous mutants. Mitoballs were not formed in 39% of *miro* KO cysts (Fig. 4*F*). The remaining 61% of KO cysts showed partial clustering, with mitochondria aggregated more on one side of the nucleus (Fig. 4*F*). These data show that mitoball formation requires Milton and Miro, but knocking out Milton abolishes mitochondrial clustering more thoroughly. This suggests that Milton could accomplish some mitochondrial trafficking independently of Miro during spermatogenesis.

Besides kinesin-based mitochondrial trafficking toward the plus end of microtubules, Milton and Miro have also been shown to influence mitochondrial mobility toward the minus end of microtubules via dynein in *D. melanogaster* and human cells, although the interactions between Milton, Miro, and the dynein complex are less well characterized (28–30). To identify the motor protein required for mitoball formation, we generated KO cysts for *khc*, kinesin light chain (*klc*) and dynein heavy chain (*dhc64c*), respectively. Mitoball formation was abolished in *khc* KO clones, although partial mitochondrial aggregations assembling those found in the *miro* KO cysts were observed in some clones (Fig. 4*G*). On the other hand, knocking out the *klc*, which is dispensable for Milton-Miro-Khc-mediated mitochondrial trafficking in axons (23), had little impact on mitoball formation (Fig. 4*G*). Similarly, in *dhc64c* KO clones, mitoball-like clusters still formed next to the fusome, although the spermatocytes were organized more in a rosette in some cysts, with all the 16 mitoballs located in the center (*SI Appendix*, Fig. S4*F*). Based on these data, we conclude that Milton-mediated mitoball formation relies primarily on Khc.

### Mitoball-Free Mutants Show Reduced Male Fertility and Carry Swollen Mitochondria in Their Spermatocytes.

Since completely knocking out *milton* is lethal at the organismal level, we designed a number of guide RNAs to generate hypomorphic alleles that are homozygous viable, as they allow us to examine the impact of Milton and mitoball formation on sperm development and male fertility. There are four protein isoforms of Milton in *D. melanogaster*, which differ at their N terminus but are identical from the Khc-binding region onwards. One of the large isoforms (Milton-A) is 1,166 amino acids (AA) long (Fig. 4*D*). By designing guide RNAs targeting different regions of *milton*, we found that terminating Milton upstream of the AA840 was lethal (e.g., *milt^KO3^* and *milt^KO4^*, *SI Appendix*, Fig. S4*B*), while mutants that carried premature stop codons or in-frame deletions after that were homozygous viable. We examined mitoball formation in three such mutants: *milt^850Ter^*, *milt^948Ter^*, and *milt^Δ904-945^*. *milt^850Ter^* flies carried a point mutation at AA850, which transforms the Glu*^850^* to a premature stop codon (*SI Appendix*, Fig. S4*B*). They completely lacked mitoballs (Fig. 4*H*), just like the *milton* KD or KOs (Fig. 4 *B* and *C* and *SI Appendix*, Fig. S4*C*). Their nebenkerns, however, appeared normal based on confocal images (*SI Appendix*, Fig. S4*E*). We also generated *milt^850Ter^*/*milt^KO1^* transheterozygotes, which were viable, and confirmed that mitoballs were absent while nebenkerns were present in these flies (*SI Appendix*, Fig. S4*G*). In contrast, *milt^948Ter^* flies, which carried a nucleotide deletion at AA946 that generates a premature stop codon at AA948, had mitoballs (*SI Appendix*, Fig. S4 *B* and *H*). Similarly, *milt^Δ904-945^* flies, which had an in-frame deletion that removes the AA904 to 945 region without affecting the rest of the C terminus, also had mitoballs (*SI Appendix*, Fig. S4 *B* and *H*). Therefore, the AA850-903 region of Milton is essential for mitoball assembly.

Leveraging the mitoball-free *milt^850Ter^* mutant, we examined the impact of losing mitoballs on male fertility. At 25 °C, the number of progeny produced by *milt^Δ904-945^* and *milt^948Ter^* homozygous males, which have mitoballs, was similar to the respective heterozygous controls (Fig. 4*I*). *milt^850Ter^* homozygous males were also fertile and gave rise to viable adults, but they produced ~23% fewer progeny than the heterozygous controls (Fig. 4*I*). On the other hand, the female fertility of *milt^850Ter^* homozygotes was comparable to controls (*SI Appendix*, Fig. S4*H*). Thus, the loss of mitoballs is associated with reduced male fertility.

Since mtDNA replication is a prominent feature of the mitoball stage, we tested whether the reduced male fertility is due to compromised mtDNA replication in flies lacking mitoballs. We stained *milt^850Ter^* testes with anti-dsDNA antibodies and counted the mtDNA nucleoids in spermatocytes from premitoball to postmitoball stages based on the nuclear sizes (Fig. 1*D*). We found that *milt^850Ter^* spermatocytes had similar mtDNA copy numbers to the wild-type cells at the corresponding stages (Fig. 1*H* and *SI Appendix*, Fig. S4*I*). Hence, mitoball formation is not necessary for mtDNA replication in the premeiotic growth phase.

We next examined whether the mitoball functions to eliminate dysfunctional mitochondria and thus improve mitochondrial quality for the later stages of sperm development. To this end, we expressed mito-QC (mCherry-GFP-Fis1) in wild-type flies to probe mitophagy (31, 32), a process that removes damaged mitochondria through autophagy. The mitochondria of the mito-QC line fluoresce both red and green under the steady state. Upon mitophagy, as mitochondria are delivered to lysosomes, mCherry fluorescence remains stable whereas GFP fluorescence becomes quenched by the acidic environment. This results in the appearance of mCherry-only foci that can be quantified as an index of cellular mitophagy. We found that the numbers of mCherry-only foci remained low and comparable from premitoball to postmitoball stages (*SI Appendix*, Fig. S4*J*), indicating no increased mitochondrial degradation at the mitoball stage.

The subcellular distribution of mitochondria could affect energy production and metabolic status (33, 34). We thus measured the adenosine triphosphate (ATP) level of testes by a quantitative bioluminescence assay and found it was significantly lower in *milt^850Ter^* mutants compared to the controls (Fig. 4*J*). This finding raised the question of whether mitochondrial function was compromised in spermatocytes with no mitoballs. As there are no efficient methods nor good markers to assess mitochondrial respiratory status at a single-cell resolution in tissues, we examined mitochondrial morphology by electron microscopy imaging. Interestingly, we found that ~33% of spermatocyte cysts in *milt^850Ter^* testes had strikingly swollen mitochondria, which were not observed in wild-type testes (Fig. 4*K*). The width of these swollen mitochondria was ~463 ± 93 nm, whereas wild-type mitochondria at a similar stage had a width of 304 ± 46 nm (*SI Appendix*, Fig. S4*K*). In addition, the individual swollen mitochondria were surrounded by a single layer of ER-like membrane and the overall ER network was more truncated (Fig. 4*K*) than the wild-type controls (Figs. 1*E* and 3*B*). Furthermore, these cysts showed an increase in mCherry-only foci when mito-QC is expressed, suggesting that the swollen mitochondria were either being degraded by mitophagy, or the cytoplasmic pH of these cysts was lower than that in controls, so more GFP fluorescence was quenched (Fig. 4*L* and *SI Appendix*, Fig. S4*L*).

It is surprising that disrupting mitochondrial trafficking could have such a profound effect on the mitochondrial volume and its interactions with other organelles. Mitochondrial swelling often indicates the opening of the mitochondrial permeability transition pore and is a hallmark of mitochondrial dysfunction. The increase in matrix volume could compromise K^+^ and Ca^2+^ fluxes, and lead to cristae remodeling and ultimately cell death (35, 36). We only observed cysts with swollen mitochondria in premeiotic stages, so it is possible that these cysts did not progress to the later stages of spermatogenesis, and the spermatids with nebenkerns we observed in *milt^850Ter^* testes (*SI Appendix*, Fig. S4 *E* and *G*) were derived from healthy spermatocytes with normal mitochondrial networks. It could also be that only spermatocytes in certain developmental points are particularly sensitive to mitochondrial-trafficking disruption and will show the mitochondrial swelling phenotype. Further studies are required to gain a better understanding of how mitoballs and Milton modulate mitochondrial morphology, function, and turnover to support sperm development.

## Discussion

This study characterized premeiotic mitochondrial clusters formed in insect spermatogenesis. We showed that mitoball-like structures were transient organizations formed in a wide range of insect species. In *D. melanogaster*, mitoballs assembled and dissembled in prophase I, and they contained other organelles including ER, Golgi bodies and the fusome. Microtubule-dependent mitochondrial trafficking was essential for mitoball formation as knocking out the adapter proteins Milton and Miro that link mitochondria to motor proteins abolished mitoball formation. Loss of mitoball was associated with reduced male fertility, suggesting its role in sperm development and potency.

The mitoball of the male germline resembles the Balbiani body of the female germline. The Balbiani body is a cytoplasmic aggregation enriched with mitochondria. It is found in the oocytes of many animal species including insects and vertebrates (21, 37). Like the mitoball, the Balbiani body is a transient structure formed in early meiosis, and it contains other organelles such as ER and Golgi bodies (37–40). Furthermore, the Balbiani body in *D. melanogaster* also depends on Milton-mediated mitochondrial trafficking (28). However, the development of the *Drosophila* Balbiani body in oocytes relies on the transportation of mitochondria via the fusome from the rest of cyst cells and is blocked by mutations in *hts*, whereas mitoball formation does not rely on *hts* or an intact fusome (Fig. 3*D*). It is also unclear whether the mitoball is rich in proteins and RNAs, and organized by functional amyloids into a dense matrix that sequesters mitochondria and other cellular components like the *Xenopus* Balbiani body (37, 41).

Nevertheless, the similarities between the mitoball and the Balbiani body suggest that they may serve common functions in germ cell development. Since the discovery of the Balbiani body in the 19th century, it has been suggested to play roles in a number of processes including the localization of organelles and macromolecules to the germplasm, lipogenesis, and the selection/elimination of dysfunctional mitochondria from female germline cells, although evidence for these suggested roles is sparse (37, 42). Our study shows that the mitoball in *D. melanogaster* does not operate to eliminate dysfunctional mitochondria in early spermatogenesis as mitophagy-mediated mitochondrial degradation was not up-regulated at the mitoball stage (*SI Appendix*, Fig. S4*J*). Instead, we found mitoball-free testes had reduced ATP levels, and they carried cysts with abnormally swollen mitochondria that might be undergoing mitophagy (Fig. 4 *J*, *K*, and *L*). This suggests that mitochondrial trafficking and distribution could play a role in shaping mitochondrial morphology, function, and turnover, which allows efficient energy and metabolite production during the spermatocyte growth phase to support subsequent development. Mitochondrial movement could modulate mitochondrial function directly, or indirectly by affecting the distributions of other components as the mitoball might act as a scaffolding hub that assembles other organelles and germplasm components to facilitate certain biological processes that support mitochondrial function and sperm development. It is worth exploring whether mitochondrial swelling is a male germ-cell-specific defect associated with the *milt^850Ter^* mutation or also occurs in other tissues.

We showed that Milton and Miro mediated mitoball formation. We further deduced that a 54 AA region in the Milton C terminus was essential for this process. Milton and Miro are part of a conserved protein complex and function together to regulate mitochondrial transport in various species, including humans. However, polarized mitochondrial clustering was more clearly abolished when we knocked out *milton* than *miro,* suggesting that Milton can regulate mitochondrial trafficking independently of Miro during spermatogenesis. Similarly, a recent study showed that Milton and Miro affected mitochondrial transport in *D. melanogaster* bristle cells differently, with Milton being required for antegrade transport and Miro being needed for retrograde mobility (29). Recently, the human Milton proteins have also been found to form functional anterograde mitochondrial transport complexes in the absence of Miro (43). How Milton operates independently from Miro is unclear. Milton has neither a mitochondrial import sequence nor a transmembrane domain. Its interaction with mitochondria is via binding to Miro, which is a mitochondrial outer membrane protein. However, in a previous study, the C terminus of *D. melanogaster* Milton (AA847-1,116), which does not bind to Miro, was shown to localize to mitochondria when expressed in COS7 cells. Hence, it has been proposed that the C-terminal region could tether Milton to mitochondria, likely through interacting with another mitochondria-anchoring protein (23). The 54 AA region essential for mitoball formation (AA850-903) lies in the C terminus of Milton, suggesting that it could be the domain that mediates the Miro-independent interactions between Milton and mitochondria and thus allows Milton to regulate mitochondrial transport in the absence of Miro.

Humans have two *milton* orthologs TRAK1 and TRAK2, which mediate microtubule-based mitochondrial trafficking in a similar manner. The function of TRAK1 and TRAK2 have been characterized in neurons and some other cell types (24, 27), but not in spermatogenesis. In *D. melanogaster* spermatogenesis, besides playing a role in mitoball assembly, Milton is also known to mediate mitochondrial elongation and microtubule sliding during spermatid development (44, 45). In mutant clones completely lacking Milton or Miro, spermatid elongation was slowed and arrested prematurely (44, 45). As a hypomorphic mutant, *milt^850Ter^* flies had nebenkerns and did not show obvious abnormalities in mitochondrial elongation or premature arrest of spermatid elongation (*SI Appendix*, Fig. S4*M*). However, due to difficulties in measuring the speed of mitochondrial and spermatid elongation across different testes within and between different genotypes, we cannot rule out the possibility that the reduced male fertility of *milt^850Ter^* flies is a combined effect of the lack of mitoballs and minor defects in spermatid development. Further investigation is needed to examine the impact of the *milt^850Ter^* mutation in the later stages of sperm development in *D. melanogaster*.

Sperm quality, which is essential for sexual reproduction, is under intense selection. Sperm morphogenesis is one of the most fast-evolving features in the animal kingdom (46, 47). Structures and proteins that are not necessary will be eliminated quickly so that all the resources are dedicated to the sperm’s performance. Hence, the fact that we observed premeiotic mitochondrial clusters in spermatocytes of most of the insect species suggests that the mitoball could be a common mechanism to maintain sperm quality. Some species, however, do not seem to form premeiotic mitochondrial clusters. This is particularly surprising for *D*. *bocqueti* and *A. coluzzii* because their closely related species, *D. serrata* and *A. stephensi*, form clear mitochondrial clusters in spermatocytes (Fig. 2 *C* and *D*). Assuming that the mitoball stage was not absent in our samples because it is particularly short-lasting in those species, it raises the question of alternative mechanisms for spermatogenesis which do not involve the mitoball as an evolutionary strategy. It is worth examining more species to gain a broader perspective on the evolutionary divergence of the premeiotic mitochondrial clustering in the animal kingdom. This will reveal whether the mitochondrial clustering in spermatocytes is also conserved in vertebrates, like the Balbiani body, and whether there is evolution pressure to select for mitochondrial clustering for male germ cell development in certain lineages.

In summary, this study provides unique insights into the dynamic nature of the mitochondrial network during insect spermatogenesis. It serves as a foundation for future characterizations of premeiotic mitochondrial clustering and how it impacts mitochondrial function and sperm development. This work also encourages investigations of Milton’s role in human mitochondrial function and male fertility, given its conserved role in mitochondrial trafficking.

## Materials and Methods

### *Drosophila* Husbandry and Stocks.

All *D. melanogaster* stocks were raised on standard media at 25 °C unless otherwise stated. *D. melanogaster* stocks and other *Drosophila* and insect species used in this study are listed in *SI Appendix*, Tables S1–S3. For the *milton* and *miro* mutants generated by CRISPR-Cas9-based editing in this study (*milt^KO1^*, *milt^KO2^*, *milt^KO3^*, *milt ^KO4^*, *milt ^850Ter^*, *milt ^Δ904-945^*, *milt^948Ter^*, *miro^KO1^*, and *miro^KO2^*), gRNAs were designed using FlyCRISPR target finder (https://flycrispr.org) and cloned into a pCFD5 plasmid. Plasmids were injected into *nos-Cas9* embryos to establish individual stocks. The nature of mutations was verified by Sanger sequencing. All primers and guide RNAs used are listed in *SI Appendix*, Table S4.

### Immunofluorescence Imaging by Confocal.

Dissected testes were fixed in phosphate buffered saline (PBS) with 4% paraformaldehyde (pH 7.4) for 30 min, washed three times in PBS, and permeabilized with PBS containing 0.1% TritonX-100 (PBST) for 1 h at room temperature. The samples were then blocked in PBST supplement with 3% bovine serum albumin for 1 h. If subsequent immunostaining was required, testes were incubated with primary antibodies overnight at 4 °C, followed by another overnight incubation with secondary antibodies. Some samples were incubated with phalloidin Alexa Fluor 647 (1:200, ThermoFisher Scientific, A22287) at the secondary antibody staining step. Samples were then mounted in VECTASHIELD with DAPI (Vector Laboratories, H-1200) and imaged on a Leica SP8 confocal microscope. For *PolG1*-*Halo* flies, the testes were incubated with 0.5 μM HaloTag TMR ligand (1:200, Promega, G8251) for 2 h at room temperature, mounted and imaged as described above.

The primary antibodies used for immunofluorescence staining include mouse anti-ATP5A (1:1,000, Abcam, ab14748), mouse anti-dsDNA (1:200, Abcam, ab27156), mouse anti-α-spectrin [1:20, Developmental Studies Hybridoma Bank (DSHB), 3A9], and mouse anti-α-tubulin (1:20, DSHB, 12G10). The secondary antibodies used include goat anti-mouse IgG Alexa Fluor 488 (1:200, ThermoFisher Scientific, A-11029), goat anti-mouse IgG Alexa Fluor 594 (1:200, ThermoFisher Scientific, A-11005), and goat anti-mouse IgG Alexa Fluor 647 (1:200, ThermoFisher Scientific, A-21235).

### EdU Staining.

Testes were dissected in Schneider’s media (ThermoFisher Scientific, 21720024) and incubated in Schneider’s media supplemented with 30 μM of EdU (5-ethynyl-2′-deoxyuridine) for 2, 4, or 16 h in the dark at room temperature. The samples were then fixed and permeabilized as described above. EdU detection was performed with the Click-iT EdU 647 Cell Proliferation Kit (ThermoFisher Scientific, C10340) in accordance with the manufacturer’s instructions. Samples were mounted and imaged on a Leica SP8 confocal microscope.

### Time-Lapse Confocal Imaging.

Testes were dissected from 1 to 2-d-old bamGAL4 > UAS-mito-Dendra2 males at room temperature and immediately transferred with a drop of Schneider’s media to a glass slide. The coverslip was gently placed onto the top of the testes to allow a small volume of Schneider’s media to be retained during imaging. The edges of the glass slide were then sealed with nail polish, and samples were imaged on the Leica SP8 confocal. If photo conversion was required, an area of interest was selected and illuminated with a UV laser (405 nm) for 10 s until all the Dendra2 had been converted from the green to the red form. The samples were then imaged with 488-nm and 561-nm lasers to detect green and red fluorescence signals, respectively.

### Electron Microscopy Imaging.

Samples were fixed in the fixative media (2% glutaraldehyde/2% formaldehyde in 0.05 M sodium cacodylate buffer pH 7.4 containing 2 mM CaCl_2_) overnight at 4 °C. After washing five times with 0.05 M sodium cacodylate buffer (pH 7.4), samples were osmicated (1% osmium tetroxide, 1.5% potassium ferricyanide, 0.05 M sodium cacodylate buffer pH 7.4) for 3 d at 4 °C. After washing five times in distilled H_2_O (dH_2_O), samples were treated with 0.1% (w/v) thiocarbohydrazide for 20 min at room temperature in the dark. After washing five times in dH_2_O, samples were osmicated a second time for 1 h at room temperature in 2% osmium tetroxide. Samples were washed five times in dH_2_O, and block stained with uranyl acetate (2% uranyl acetate in 0.05 M maleate buffer, pH 5.5) for 3 d at 4 °C. Samples were washed again five times in dH_2_O and then dehydrated in a graded series of ethanol (50/70/95/100/100% dry), 100% dry acetone and 100% dry acetonitrile, three times in each for at least 5 min. Samples were infiltrated with a 50/50 mixture of 100% dry acetonitrile/Quetol resin [without benzyl dimethylamine (BDMA)] overnight, followed by 3 d in 100% Quetol (without BDMA). Samples were then infiltrated for 5 d in 100% Quetol resin with BDMA, exchanging the resin each day. The Quetol resin mixture contains 12 g Quetol 651, 15.7 g nonenyl succinic anhydride, 5.7 g methyl nadic anhydride and 0.5 g BDMA. Samples were placed in embedding molds and cured at 60 °C for 3 d. Ultrathin sections (~70 nm) were cut using a Leica Ultracut microtome and mounted on melinex plastic coverslips. The coverslips were mounted on aluminum EM stubs using conductive carbon tabs and the edges of the slides were painted with conductive silver paint. Then, samples were sputter coated with 30-nm carbon using a Quorum Q150 T E carbon coater. Samples were imaged in a Verios 460 scanning electron microscope (FEI/ThermoFisher Scientific) at 4 keV accelerating voltage and 0.2 nA probe current in backscatter mode using the concentric backscatter detector in immersion mode at a working distance of 3.5 to 4 mm; 1,536 × 1,024 pixel resolution, 3 us dwell time, 4 line integrations.

### The Candidate RNAi/KO Screen.

For the RNAi screen, males carrying the UAS-RNAi constructs were crossed to females carrying both nosGAL4 and bamGAL4 at 29 °C to allow early and strong expression of RNAi. Testes were dissected from ten 1 to 2-d-old male progeny and stained with anti-ATP5A antibodies and DAPI for immunofluorescence imaging as described above.

For KO mutants, 10 to 15 testes were dissected from males if the mutant was homozygous viable and prepared for immunostaining. If the mutant was not homozygous viable. Germline KO clones/cysts were generated in heterozygous mutants. To generate germline KO clones, flies carrying both the mutant allele and an FRT site on the same arm of the chromosome were generated by recombination through genetic crosses. The FRT-mutant flies were then crossed to flies with a heat-shock inducible or germline-driven flippase (hs-FLP or nosGAL4 > UAS-FLP), the same FRT site, and a nuclear targeted fluorescent protein marker, which was present in heterozygous mutant clones and wild-type clones, but not in homozygous mutant clones. Genetic crosses were performed at 25 °C in vials. If the hs-FLP was used, the vials were shifted to 37 °C (heat shock treatment) for 2 h each time for 3 consecutive days once the progeny reached the wandering 3rd instar larval stage. Testes were dissected from 1 to 2-d-old male progeny and prepared for immunofluorescence staining as described above. The FRT/FLP lines used in this study are listed in *SI Appendix*, Table S3.

### Image Analyses and Quantifications.

The nuclear and mitoball diameters were measured using the Leica LAS X software. In total, 28 spermatocytes from testes of at least five different genotypes were measured for each developmental stage. The mitochondrial movement was quantified based on time-lapse imaging of bamGAL4 > mito-Dendra2 testes. Images were taken every 2 s for 10 min. In total, 48 mitochondria, across 5 different mitoballs that remained within the focal plane, were tracked. The distance moved by a given mitochondrion between individual frames was calculated by using the Leica “draw scale bar” tool. The total distance moved by each mitochondrion was divided by the total time between the frames to obtain the average speed in nms^−1^.

To quantify mtDNA stained by anti-dsDNA antibodies, a series of Z-stack images were acquired at 1-μm steps to cover the entire cell. The number of puncta was counted using the Leica “draw counter” feature. Data points in Fig. 1*H* and *SI Appendix*, Fig. S4*I* show the number of dsDNA puncta per cell for four cells at each developmental stage, where all the cells at a given developmental stage were from different testes.

To quantify mitoQC, the number of red (mCherry signal) and green (GFP signal) puncta of 0.2 to 0.5 μm in diameters were counted in cells at the respective developmental stage using an in-house FIJI plugin created by Dr Richard Butler at the Gurdon Institute, University of Cambridge. To obtain the numbers of mCherry-only puncta in each cell, the numbers of green puncta were subtracted from the numbers of red puncta.

### Fertility Test.

Prior to the fertility assays, males of *milt^850Ter^, milt^Δ904-945^*, and *milt^948Ter^* were crossed to *w1118* females with balancer chromosomes to establish *milton* mutants with an isogenic nuclear (except for the 2nd chromosome) and mitochondrial background (i.e., *w1118* mtDNA). For the male fertility assay, individual 1 to 2-d-old males were mated with three *w1118* virgin females at 25 °C. For each genotype, the total number of progeny was counted for 20 males. For female fertility assays, four 1 to 2-d-old females were mated with three young *w1118* males at 25 °C, and the total number of progeny was counted for each cross. For each genotype, 20 replicates were performed.

### ATP Assay.

ATP levels were measured using the ATP determination kit (ThermoFisher Scientific, A22066). For each genotype, 10 testes were dissected from 1 to 2-d-old males and homogenized in 100 μL of 6 M guanidine HCl (ThermoFisher Scientific, 24115) and 0.01 M Tris-HCl (pH 7.3), and frozen in liquid nitrogen for 5 min. The sample was then incubated at 95 °C for 5 min and centrifuged at 12,000 × *g* for 10 min at 4 °C. 10 μL of the sample was used for each 100 μL reaction as instructed in the kit manual. ATP levels for each sample were normalized to protein concentration measured using the Pierce BCA protein assay kit (ThermoFisher Scientific, 23225). For each genotype, six replicates were performed.

### Statistical Analyses.

Statistical analyses were performed using R (version R 4.0.5) and graphs were plotted using Prism 9 (GraphPad). All comparisons of means for the mitochondrial genome quantification, normalized proportion of mCherry-only foci and female fertility were performed using one-way (ANOVA) with Tukey’s honestly significant difference post hoc test. For the male fertility and mitochondrial width data, the Mann–Whitney *U* test was used as the data were not normally distributed. Student’s *t* test was used to compare the means for the ATP assay. For all statistical tests, key assumptions were formally tested in R, and the datasets were deemed suitable. Statistical significance was defined by ns (not significant) *P* ≥ 0.05, *P < 0.05, **P < 0.01, and ***P < 0.005.

## Supplementary Material

Appendix 01 (PDF)

Movie 1. **A Time-lapse movie showing the dynamics of mitochondria within mitoballs**. The testis was dissected from bamGAL4 > mito-Dendra2 flies cultivated in Schneider’s media. The movie lasts 15 min 30 s in real time with images taken every 30 s. Scale bar: 10 μm

Movie 2. **A 3D projection of Z-stack images displaying the positions of the fusome and mitoballs in a cyst**. The testis was dissected from sqh-mito-YFP (green) flies, and stained with DAPI (blue) and anti-α-spectrin antibodies (magenta).

Movie 3. **A series of Z-stack images covering the entire depth of a testis tip expressing milton RNAi in early spermatogenesis driven by nosGAL4 and bamGAL4**. The testis was stained with DAPI (blue) and anti-ATP5A antibodies (green). Scale bar: 100 μm

## Data Availability

All study data are included in the article and/or supporting information. All fly stocks will be made available upon request.
