# Supplementary material for: Milton assembles large mitochondrial clusters, mitoballs, to sustain spermatogenesis
Source: Proc Natl Acad Sci U S A. 2023 Aug 14;120(34):e2306073120. doi: 10.1073/pnas.2306073120 (PMC10450580; doi:10.1073/pnas.2306073120)
Supplement: Supplementary file 1 — Appendix 01 (PDF) [file pnas.2306073120.sapp.pdf]

Supplementary Materials

The PDF file includes

Figures S1 to S4
Tables S1 to S4
Movie legends for Movies 1 to 3

Other supplementary Materials for this manuscript include the following:

Movies 1 to 3

Supplementary Figures

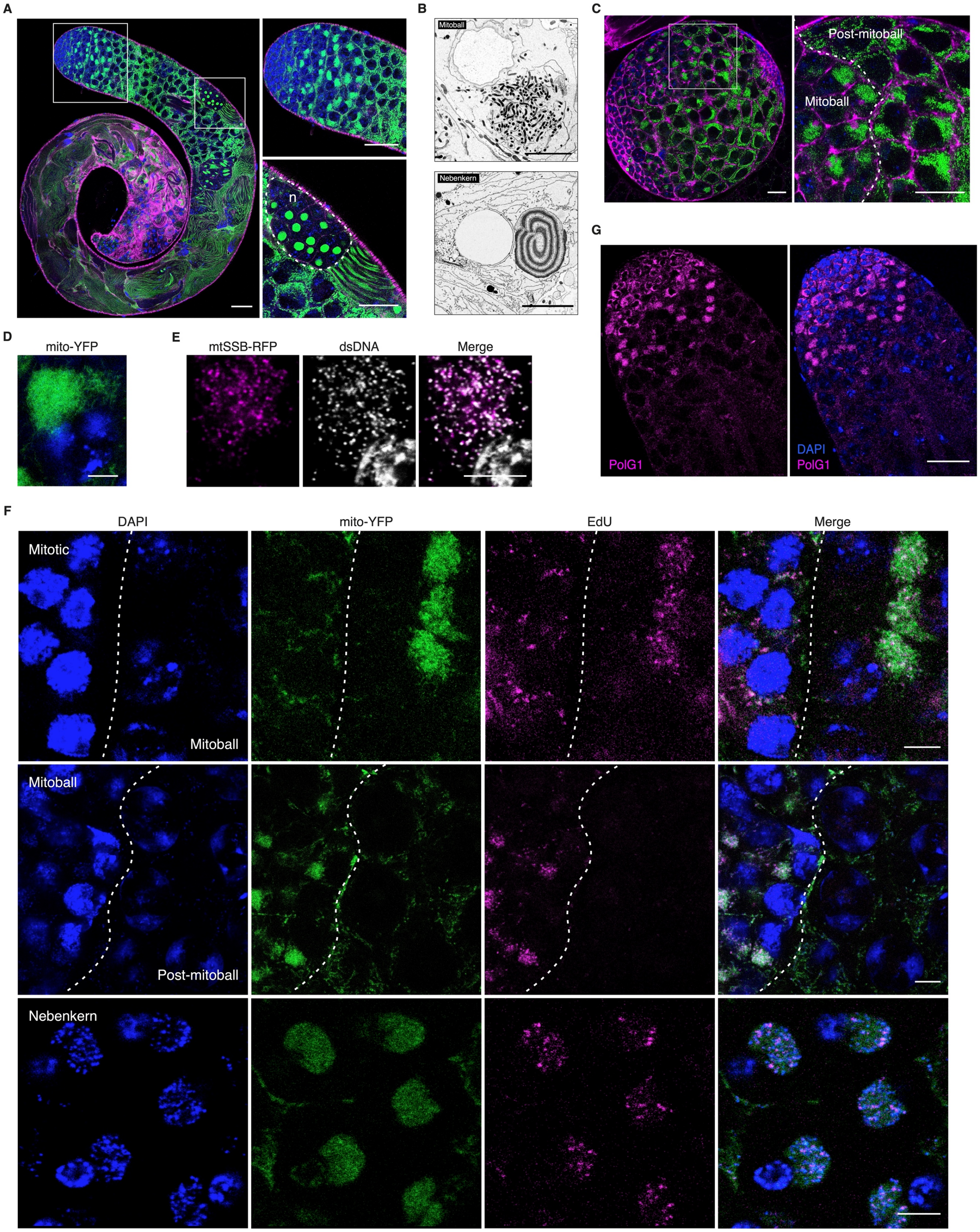

Figure S1: Mitochondrial dynamics and mtDNA replication during *D. melanogaster* spermatogenesis. A) A tiled confocal image of an adult *D. melanogaster* testis illustrating mitochondrial dynamics at different stages of spermatogenesis, including the formation and disassembly of mitoballs in spermatocytes, the nebenkerns in the 64-cell spermatids and the elongation of mitochondrial derivatives after the nebenkern stage (zoomed-in views). The testis was stained with DAPI (blue), anti-ATP5A antibodies (green), and phalloidin (magenta) to visualize DNA, mitochondria, and cell/cyst boundaries, respectively. Scale bars: 50 μm. **B)** Electron microscopic images of mitoball and nebenkern stage sperm cells. Scale bars: 5 μm. **C)** A 3rd instar larval testis stained with DAPI (blue), anti-ATP5A antibodies (green),

and phalloidin (magenta). Spermatocytes at mitoball and postmitoball stages are separated by a dotted line in the zoomed-in view. Scale bars: 25 µm. **D)** The three chromatin clumps that remain close to the inner nuclear envelope are typical for the spermatocyte nucleus in the premeiotic G2 phase (1). The sample was stained with DAPI (blue) and anti-ATP5A antibodies (green). Scale bar: 5 µm. **E)** A spermatocyte stained with anti-dsDNA antibodies to visualize mtDNA nucleoids. dsDNA staining gives more discrete foci in the cytoplasm (white) than mtSSB-RFP puncta (magenta), which facilitates the counting. Z-stack images that cover the entire cell were taken to generate 3D projections for counting the number of mtDNA nucleoids in spermatocytes at different stages. Scale bar: 5 µm. **F)** EdU staining (magenta) indicating mtDNA replication in mitotic spermatogonial cells, spermatocytes at the mitoball stage, postmitoball spermatocytes and spermatids at the nebenkern stage. Cysts at different stages are separated by dotted lines. Testes were incubated with EdU for 16 h and stained with DAPI (blue) and anti-ATP5A antibodies (green). Scale bars: 5 µm. **G)** The expression level of mtDNA polymerase *PolG1* in early spermatogenesis. The testis was isolated from flies with *PolG1* endogenously tagged with Halo and stained with TMR ligands (magenta) and DAPI (blue). Scale bar: 25 µm.

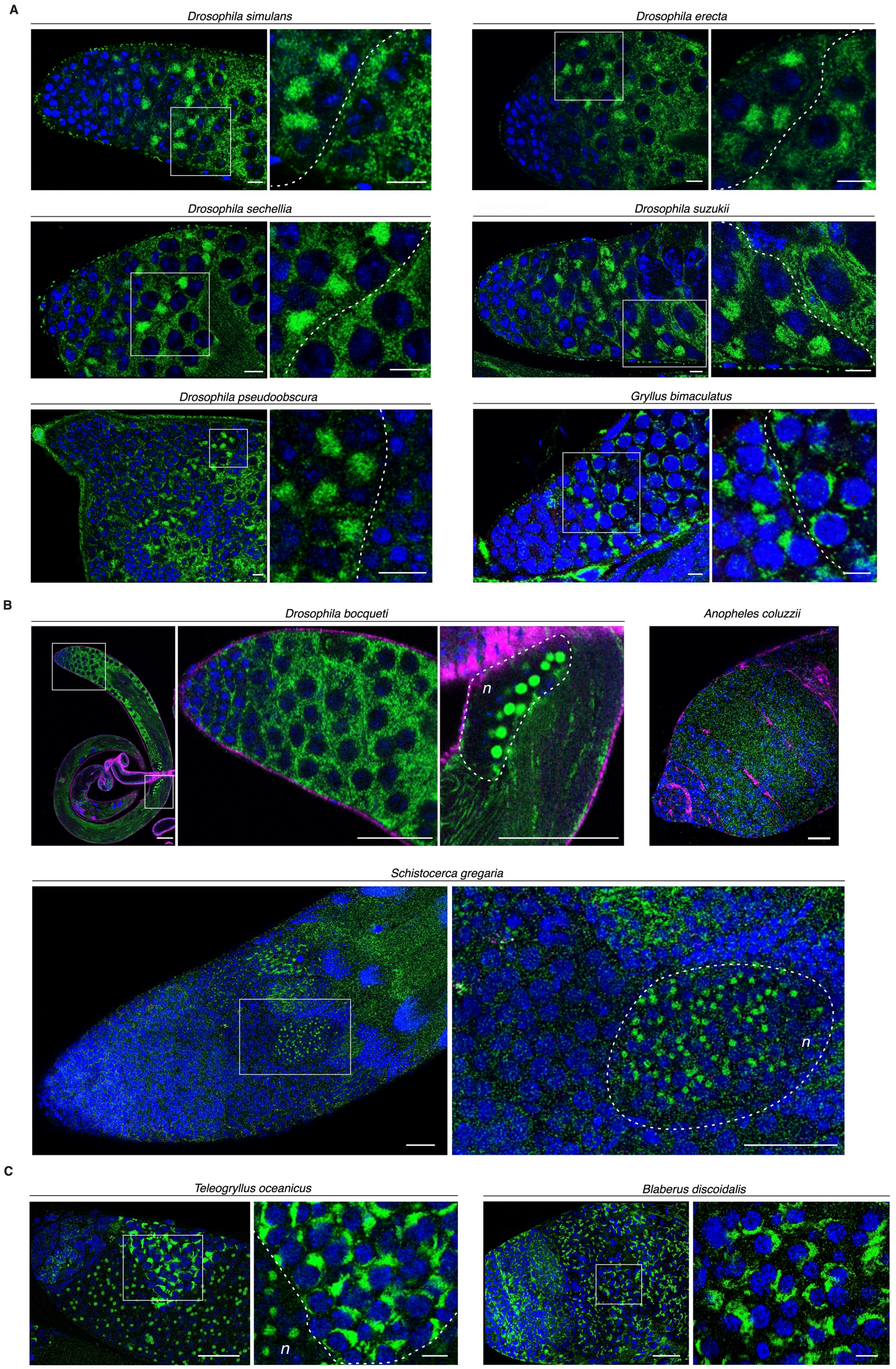

Figure S2: Premeiotic mitoballs and similar mitochondrial clusters were found in a wide range of insect species. A) Representative images of species with mitoballs in primary spermatocytes. Spermatocyte cysts at the mitoball and postmitoball stages are separated by a dotted line in the zoomed-in view. Testes were stained with DAPI (blue) and anti-ATP5A antibodies (green). Scale bars: 10 µm. **B)** Representative images of species with no premeiotic mitoball or mitochondrial clusters detected. Spermatid cysts at the nebenkern (*n*) stages are outlined in the zoomed-in view. Testes were stained with DAPI (blue), anti-ATP5A (green) antibodies, and phalloidin (magenta). Scale bars: 50 µm. **C)** Other types of premeiotic mitochondrial clusters found in *Teleogryllus oceanicus* and *Blaberus discoidalis*. Testes were stained with DAPI (blue) and anti-ATP5A (green) antibodies. A nebenkern-stage spermatid cyst is labelled as '*n*' in the zoomed-in view for *T. oceanicus*. Scale bars: 50 µm (10 µm for the zoomed-in views).

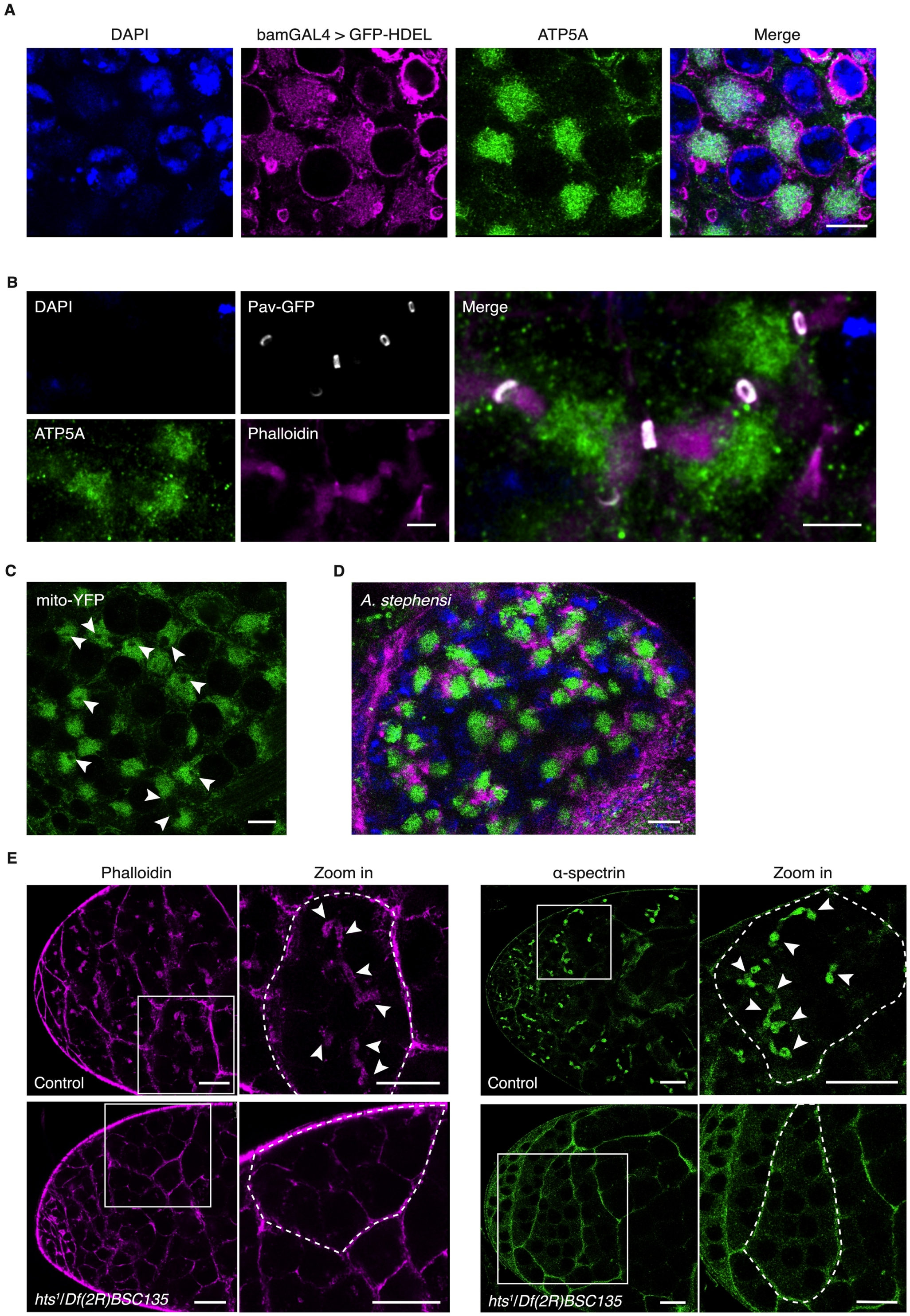

Figure S3: ***D. melanogaster*** **mitoballs are packed with ER, surrounded by Golgi bodies, and connected by the fusome. A)** The localization of the ER network visualized by expressing GFP-HDEL under bamGAL4 (magenta). The testis was stained with DAPI (blue) and anti-ATP5A antibodies (green). Scale bar: 10 μm. **B)** The relative positions of nuclei (DAPI, blue), mitoballs (anti-ATP5A antibodies, green), ring canals (Pav-GFP, white) and the fusome (phalloidin, magenta) in four spermatocytes of a cyst. Scale bars: 10 μm. **C)** The empty holes in the mitoballs (arrowheads) indicate the positions of the fusome in each spermatocyte. Mitoballs (green) were visualized by sqh-mito-YFP. Scale bar: 10 μm. **D)** Mitoballs in *A. stephensi* were located close to the fusome. The testis was stained with DAPI (blue), anti-ATP5A antibodies (green), and phalloidin (magenta). Scale bar: 10 μm. **E)** The fusome (arrowheads), visualized by staining with phalloidin (magenta) or anti-α-spectrin antibodies (green), was present in control testes but not in *hts* mutant (*hts*^{1}/*Df(2R)BSC135*). One cyst is outlined by dotted white lines in the zoomed-in views. The genotype for the control is *Df(2R)BSC135*/*CyO*. Scale bars: 25 μm.

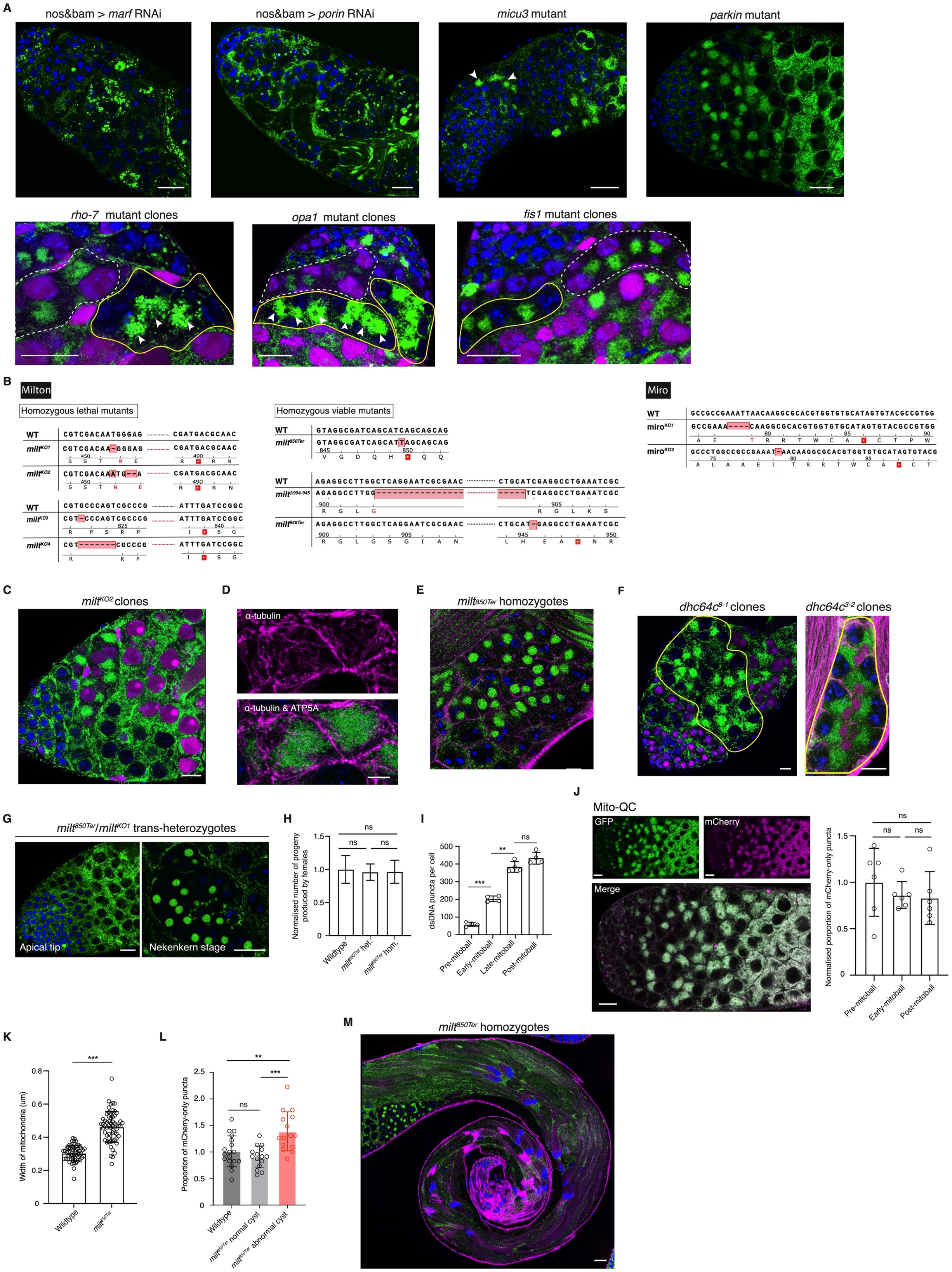

Figure S4: A candidate screen revealed that Milton is required for mitoball formation and male fertility. A) Representative images of mitochondrial organization in early spermatogenesis after knocking out/down some genes tested in the RNAi/KO screen. RNAi expression was driven by both nosGAL4 and bamGAL4. For the KO screens, testes were dissected from homozygous males if the mutant is homozygous viable (*micu3* and *parkin*), or germline KO clones were generated for mutants that are homozygous lethal (*rho-7*, *fis1* and *opa1*). Mitoballs are indicated with white

arrowheads for some images. KO cells are marked by the absence of RFP (magenta) in their nuclei. Scale bars: 25 µm. **B)** Sequences of *milton* and *miro* mutants generated in this study. **C)** Germline clones of $milt^{KO2}$ show that knocking out *milton* abolishes mitoball formation. A KO cyst and a wild-type cyst at a similar developmental stage are outlined by solid yellow and dotted white lines, respectively. Scale bar: 25 µm. **D)** Microtubule organization in the mitoball regions visualized by anti-α-tubulin antibodies (magenta). The testis was also stained with anti-ATP5A antibodies (green). Scale bar: 5 µm. **E)** Nebenkerns were present in $milt^{850ter}$ flies. The testis was stained with DAPI (blue), anti-ATP5A antibodies (green) and phalloidin (magenta). Scale bar: 10 µm. **F)** Knocking out *dhc64c* has a minor impact on mitochondrial clustering in premeiotic spermatocytes. Mutant cysts for *dhc64c* were outlined by solid yellow lines. Left panel: The testis was stained with DAPI (blue), anti-ATP5A antibodies (green) and KO cells are marked by the absence of RFP (magenta) in their nuclei. Right panel: the testis was stained with DAPI (blue), anti-ATP5A antibodies (green) and phalloidin (magenta). Scale bars: 10 µm. **G)** $milt^{850ter}/milt^{KO1}$ trans-heterozygotes have no mitoballs, but nebenkerns still form. The testis was stained with DAPI (blue) and anti-ATP5A antibodies (green). Scale bars: 20 µm. **H)** No difference in female fertility between homozygous $milt^{850ter}$ flies, heterozygous $milt^{850ter}$ flies, and the wild-type control (n = 20). Error bars: SEM, one-way ANOVA and Tukey's post hoc, ns $p>0.05$. **I)** The total mtDNA copy numbers of $milt^{850ter}$ spermatocytes are comparable to those of control cells at the corresponding stages (Fig 1H) (n = 4). Error bars: SD, one-way ANOVA and Tukey's post hoc, ns $p>0.05$, ** $p<0.01$, *** $p<0.005$. **J)** Representative images of mito-QC and quantification of the mCherry-only puncta from premitoball to postmitoball stages for wild-type flies. Mito-QC flies show both mCherry (magenta) and GFP (green) fluorescence under the steady state. mCherry-only foci larger than 0.5 µm were not observed in germ cells in cysts, but in somatic cells surrounding individual cysts. Scale bars: 20 µm. For individual germ cells, the proportion of mCherry-only puncta was calculated by subtracting the number of GFP puncta from mCherry puncta and dividing by the total number of puncta (n = 6). Error bars: SD, one-way ANOVA and Tukey's post hoc, ns $p>0.05$. **K)** The width of mitochondria measured from electron microscopy micrographs (n = 60). Error bars: SD, Mann–Whitney *U* test, *U* test, *** $p<0.005$. **L)** The proportion of mCherry-only puncta in spermatocytes of wild type and $milt^{850ter}$ flies expressing mito-QC (n = 15). Error bars: SD, one-way ANOVA and Tukey's post hoc, ns $p>0.05$, ** $p<0.01$, *** $p<0.005$. **M)** A confocal image illustrating the spermatid development and morphology in the $milt^{850ter}$ mutant. Scale bar: 25 µm.

Supplementary Tables 1 - 4

Table S1. A list of genes knocked down using RNAi to determine their effect on the mitoball phenotype. Related to Fig 4A.

Gene (CG number)	RNAi lines (BDSC or VDRC)	Mitoball and other testis phenotypes
**Mitochondrial dynamics**		
Drp1 (CG3210)	27682	No effect
Fis1(CG17510)	6307	No effect
larp (CG42551)	42578	No effect
Marf (CG3869)	Ming Guo's lab	Abnormal mitochondrial network, no mitoballs
milt (CG43227)	44477	No mitoballs
Mitofilin (CG6455)	63994; 43245	No effect
Opa1 (CG8479)	32358	No effect
park (CG10523)	38333	No effect
rho-7 (CG8972)	35617	No effect
**Cell polarity**		
aPKC (CG42783)	34332; 35140; 38245	No effect
baz (CG5055)	38213; 39072	No effect
crb (CG6383)	34999; 38373; 27697	No effect
Kr-h2 (CG9159)	67290	No effect
par-1 (CG8201)	32410; 35342	No effect
par-6 (CG5884)	39010; 38361	No effect
Patj (CG12021)	26282; 35747; 38193	No effect
sdt (CG32717)	33909; 38988; 37510	No effect
**piRNA**		
arx (CG3893)	v40480	No effect
aub (CG6137)	35201; 39026; 33728	No effect
cuff (CG13190)	35182	No effect
del (CG9252)	32375	No effect
mael (CG11254)	v100907& v313155	No effect
piwi (CG6122)	33724; v101658 & v313199	No effect
rhi (CG10683)	35171	No effect
zuc (CG12314)	35228; 35227; 36742	No effect
**mtDNA replication**		
mtDNA-helicase (CG5924)	31079; 31080	No effect
mtSSB (CG4337)	50600	No effect
PolG1 (CG8987)	31081; 31098; v106955	No effect
PolG2 (CG33650)	67925; 51158	No effect
TFAM (CG4217)	26744; 57742	No effect
Top3α (CG10123)	44451	No effect
**Specific/High expression in testis**		
CG10344	42505; 34877	No effect
CG14708	53020	No effect
CG14840	33333, 62471	No effect
CG14841	44059	No effect
CG15357	58156	No effect
CG30161	41977	No effect
CG33252	43849	No effect
CG9641	36839	No effect
ctp (CG6998)	44044	No effect
Flo2 (CG32593)	40833; 55212	No effect
lola (CG12052)	26714	No effect
Myo10A (CG43657)	41691	No effect
Rtnl2 (CG1279)	58208	No effect
smt3 (CG4494)	28034; 36125	No effect
tau (CG45110)	28891; 40875	No effect
Tsp33B (CG14936)	39043	No effect
tsr (CG4254)	65055	No effect
VhaPPA1-2 (CG7026)	65217	No effect
vig (CG4170)	35183; 35184	No effect
**Spermatogenesis and mitochondria related**		
Afg3l2 (CG6512)	50524; 34343	No effect
Atg7 (CG5489)	27707; 34369	No effect

ATPsynβ (CG11154)	28056; 27712	No effect
ATPsynγ (CG7610)	50543	No effect
CG10749	62229	No effect
chico (CG5686)	36788; 36665	No effect
COX5A (CG14724)	58282	No effect
DCTN1-p150 (CG9206)	27721	No effect
Debcl (CG33134)	27083	Abnormal testis but mitoballs normal
djl (CG1984)	61909	No effect
dlp (CG32146)	34091; 50540	No effect
EndoG (CG8862)	55228; 62903	No effect
Exd2 (CG6744)	v103374	No effect
Hip14 (CG6017)	31591; 35012	No effect
Hsp60B (CG2830)	66328	No effect
Hsp60C (CG7235)	67003	No effect
HtrA2 (CG8464)	28544; 55165	No effect
iPLA2-VIA (CG6718)	36129	No effect
Klp67A (CG10923)	35606; 62383	No effect
knon (CG7813)	62301	No effect
kug (CG7749)	40888	No effect
Lis-1 (CG8440)	28663; 35043	No effect
Lon (CG8798)	40162	No effect
mAcon2 (CG4706)	51359; 58074	No effect
Mer (CG14228)	28007	No effect
MICU1 (CG4495)	v49349	No effect
Msp300 (CG42768)	32377; 32848	No effect
ND-PDSW (CG8844)	29592	No effect
orb (CG10868)	64002	No effect
orb2 (CG43782)	60424; 56997	No effect
porin (CG6647)	29572; 67873	Abnormal mitochondrial network, no mitoballs
Rab11(CG5771)	27730; 42709	No effect
Rab4 (CG4921)	33757	No effect
Rae1 (CG9862)	57832; 32882	Abnormal testis & no mitoballs
Rho1 (CG8416)	32383; 27727	No effect
Scsα2 (CG6255)	64025	No effect
Sec8 (CG2095)	57441	No effect
shg (CG3722)	32904	Abnormal testis but mitoballs normal
shi (CG18102)	36921	No effect
Sprn (CG14128)	57844	No effect
sqh (CG3595)	33892	No effect
Ubc6 (CG2013)	42631	Abnormal testis but mitoballs normal
YME1L (CG3499)	51752	No effect
**Others**		
aux (CG1107)	35310; 39017	Abnormal nebenkern
CG10877	55160	No effect
CG11423	42591	No effect
CG15434	52913	No effect
fz (CG17697)	34321	No effect
fz2 (CG9739)	67863	No effect
fz3 (CG16785)	66951; 44468	No effect
fz4 (CG4626)	64990	No effect
Notch (CG3936)	27988; 28981	No effect
sgg (CG2621)	31308	No effect
Sk2 (CG32484)	36741	No effect
yki (CG4005)	34067	No effect

The table lists 105 genes screened using RNAi knockdown to determine whether they have an impact on the mitoball phenotype. The listed genes are divided into seven categories based on their biological functions. All genes are represented by their short name (symbol) for brevity. All the expressions of all RNAi constructs were driven by both nosGAL4 and bamGAL4. Testes of at least 10 male flies were stained with antibodies against ATP5A to visualize mitochondria. The genotype source is given as a BDSC number referring to the Bloomington *Drosophila* Stock Centre or VDRC Vienna *Drosophila* Resource Center number starting with 'v'.

Table S2. A list of genes tested to examine their effects on mitoball formation. Related to Fig 4A.

Gene (CG number)	Source	Function	Mitoball and mitochondrial phenotype
**Homozygous Viable Mutants**			
Exd2 (CG6744)	BDSC16578 & 36433	Mitochondrial metabolism	No effect
CG12917	BDSC29197		No effect
Pink1(CG4523)	BDSC34749	Mitochondrial mitophagy/dynamics	No effect
park (CG10523)	BDSC34747 & 51652		No effect
bol (CG4760)	BDSC11794	Spermatogenesis	No effect
mtsh (CG7795)	BDSC66385		No effect
sxc (CG10392)	BDSC7182, 3058 & 79618	O-GlcNAc transferase	No effect
del (CG9252)	BDSC15249	piRNA	No effect
MICU3	MICU3[27], Whitworth lab (2)	Mitochondrial Ca^{2+} uptake	Abnormal testis
MCU	MCU[1], Whitworth lab (2)		Abnormal testis
EMRE	EMRE[1], Whitworth lab (2)		Abnormal testis
**Homozygous Lethal Mutants (germline clones generated)**			
Opa1 (CG8479)	BDSC12188	Mitochondrial dynamics/biogenesis	Abnormal mitochondrial morphology, mitoball forms
Fis1 (CG17510)	BDSC55496		No effect
rho-7 (CG8972)	BDSC80676		Abnormal mitochondrial morphology, mitoball forms
Larp (CG42551)	BDSC11687		No effect
mtDNA helicase (CG5924)	This lab (3)	mtDNA replication	No effect
PolG2 (CG33650)	This lab (3)		No effect
Patronin (CG33130)	BDSC16647, St Johnston lab	Cytoskeleton	No effect
crb (CG6383)	$Crumbs^{11A22}$ & $Crumbs^{8F105}$, St Johnston lab		No effect
β-Spectrin (CG5870)	St Johnston lab		No effect
chb (CG32435)	St Johnston lab		No effect
WASp (CG1520)	BDSC51657		No effect
piwi (CG6122)	BDSC43637	piRNA	No effect
aub (CG6137)	BDSC8517		No effect
rhi (CG10683)	BDSC12226		No effect

All genes are represented by their short name (symbol) for brevity. To determine whether the mitoball or mitochondrial phenotype was affected by the mutant gene, the testes of at least 10 male flies were stained with anti-ATP5A antibodies and imaged using confocal microscopes. For homozygous viable mutants, the testes were dissected from homozygous males. For homozygous lethal mutants, FRT recombination was used to generate germline clones that are homozygous for the corresponding mutation. The genotype source is given as a BDSC number referring to the Bloomington *Drosophila* Stock Centre, unless otherwise stated.

Table S3. A list of lines used in this study.

Line	Source
bamGAL4 and nosGal4 (III)	Recombined in this lab
α *spectrin*$^{E2\text{-}26}$ FRT2A/TM6B	Daniel St Johnston lab, Gurdon Institute, University of Cambridge
Anopheles coluzzii	Jake Baum lab at Imperial College London
Anopheles stephensi	Jake Baum lab at Imperial College London and Andrew Blagborough lab at the Department of Pathology, University of Cambridge
Balberus discoidalis	Dr Steve Rogers, Department of Zoology, University of Cambridge
Df(2R)BSC135/CyO	BDSC9423
Drosophila bocqueti	Department of Genetics, University of Cambridge
Drosophila erecta	Department of Genetics, University of Cambridge
Drosophila pseudoobscura	Department of Genetics, University of Cambridge
Drosophila saltans	Department of Genetics, University of Cambridge
Drosophila sechellia	Department of Genetics, University of Cambridge
Drosophila serrata	Department of Genetics, University of Cambridge
Drosophila simulans	Department of Genetics, University of Cambridge
Drosophila suzukii	Department of Genetics, University of Cambridge
Drosophila yakuba	Department of Genetics, University of Cambridge
Gromphadorhina portentosa	Dr Steve Rogers, Department of Zoology, University of Cambridge
Gryllus biumaculatus	Dr Steve Rogers, Department of Zoology, University of Cambridge
Schistocerca gregaria	Dr Steve Rogers, Department of Zoology, University of Cambridge
Teleogryllus oceanicus	Dr Steve Rogers, Department of Zoology, University of Cambridge
hsFLP;; FRT2A RFPnls	Daniel St Johnston lab, Gurdon Institute, University of Cambridge
hsFLP;; FRT82B GFPnls	Daniel St Johnston lab, Gurdon Institute, University of Cambridge
hsFLP; FRT40A RFPnls	Daniel St Johnston lab, Gurdon Institute, University of Cambridge
hsFLP; FRTG13 RFPnls	Daniel St Johnston lab, Gurdon Institute, University of Cambridge
FRT40A RFPnls; nosGal4 UAS-FLP	Daniel St Johnston lab, Gurdon Institute, University of Cambridge
hts^{1}	Daniel St Johnston lab, Gurdon Institute, University of Cambridge
If/CyO; ubi-FisQC	Generated for this study
Ubi-Pav-GFP	Daniel St Johnston lab, Gurdon Institute, University of Cambridge
PolG1-Halo	This lab (4)
sqh-mt-YFP	BDSC7194
sqh-YFP-ER	BDSC7195
sqh-YFP-Golgi	BDSC7193
UAS-Dendra2	Thomas Rival lab, Aix-Marseille University
UAS-GFP-HDEL	BDSC64749
Ubi-mtSSB-RFP	This lab
Ubi-mtSSB-GFP; FRT2A	This lab
dhc64c$^{8\text{-}1}$, FRT2A	Daniel St Johnston lab, Gurdon Institute, University of Cambridge
dhc64c$^{3\text{-}2}$, FRT2A	Daniel St Johnston lab, Gurdon Institute, University of Cambridge
klc^{8ex94}, FRT2A	BDSC31997
khc$^{[27]}$, FRTG13	Daniel St Johnston lab, Gurdon Institute, University of Cambridge

Table S4. A list of primers and guide RNAs used in this study

Primer/Guide	Sequence
milton gRNA 1	GAGGGCTGGGCTGGATAACC
milton gRNA 2	CAGCTCTTCGGGGCCGCGCA
milton gRNA 3	CGTGCCCAGTCGCCCGAGCC
milton gRNA 4	CGTGTGGTGCGGATTTGATC
milton gRNA 5	TGGCTCAGGAATCGCGAACC
milton gRNA 6	GCATCGAGGCCTGAAATCGC
milton gRNA 7	ACCAGAGGCCAGATCTGGGC
milton gRNA 8	AGTTCGGCGACGACATCCAA
Sequencing primers *milton* N Fwd	CGGATTCACTGCACTGTGAGC
Sequencing primers *milton* N Rev 1	GAGGGCTGGGCTGGATAACC
Sequencing primers *milton* C fwd	CCACATTTTCGGTGAACCTCGGAC
Sequencing primers *milton* C rev 1	GATTTCAGGCCTCGATGCAGGAC
Sequencing primers *milton* C rev 2	CAACTTGTTCTCGCGTAGTGC
miro gRNA 1	CCCCGAGATTGAGAGTTGCG
miro gRNA 2	CACGTGCGCCTTGTTAATTT
Sequencing primers *miro* fwd	TACCAGACAGTTTCGGATCCGATG
Sequencing primers *miro* rev	GCACATTTTTCTGTATCACGGCCTTCAC

Movie legends

Movie 1: A Time-lapse movie showing the dynamics of mitochondria within mitoballs. The testis was dissected from bamGAL4 > mito-Dendra2 flies cultivated in Schneider's media. The movie lasts 15 min 30 s in real time with images taken every 30 s. Scale bar: 10 μm

Movie 2: A 3D projection of Z-stack images displaying the positions of the fusome and mitoballs in a cyst. The testis was dissected from sqh-mito-YFP (green) flies, and stained with DAPI (blue) and anti-α-spectrin antibodies (magenta).

Movie 3: A series of Z-stack images covering the entire depth of a testis tip expressing *milton* RNAi in early spermatogenesis driven by nosGAL4 and bamGAL4. The testis was stained with DAPI (blue) and anti-ATP5A antibodies (green). Scale bar: 100 μm

References

1. G. Cenci, S. Bonaccorsi, C. Pisano, F. Verni, M. Gatti, Chromatin and microtubule organization during premeiotic, meiotic and early postmeiotic stages of Drosophila melanogaster spermatogenesis. *J. Cell Sci.* **107**, 3521–3534 (1994).

2. R. Tufi, *et al.*, Comprehensive Genetic Characterization of Mitochondrial Ca2+ Uniporter Components Reveals Their Different Physiological Requirements In Vivo. *Cell Rep.* **27**, 1541-1550.e5 (2019).

3. A. C.-Y. Chiang, E. McCartney, P. H. O'Farrell, H. Ma, A Genome-wide Screen Reveals that Reducing Mitochondrial DNA Polymerase Can Promote Elimination of Deleterious Mitochondrial Mutations. *Curr. Biol.* **29**, 4330-4336.e3 (2019).

4. A. Klucnika, *et al.*, REC drives recombination to repair double-strand breaks in animal mtDNA. *J. Cell Biol.* **222** (2023).
